# Rapid Identification of Aphid Species by Headspace GC-MS and Discriminant Analysis

**DOI:** 10.3390/insects14070589

**Published:** 2023-06-30

**Authors:** Noura J. Alotaibi, Taghreed Alsufyani, Nour Houda M’sakni, Mona A. Almalki, Eman M. Alghamdi, Dieter Spiteller

**Affiliations:** 1 Department of Biology, College of Science, Taif University, P.O. Box 11099, Taif 21944, Saudi Arabia; n.alotaibi@tu.edu.sa; 2 Department of Chemistry, College of Science, Taif University, P.O. Box 11099, Taif 21944, Saudi Arabia; nour.h@tu.edu.sa (N.H.M.); m.awwad@tu.edu.sa (M.A.A.); 3 Chemistry Department, Faculty of Science, King AbdulAziz University, P.O. Box 80200, Jeddah 21589, Saudi Arabia; emalghamdi@kau.edu.sa; 4 Chemical Ecology/Biological Chemistry, University of Konstanz, Universitätsstraße 10, 78457 Konstanz, Germany; dieter.spiteller@uni-konstanz.de

**Keywords:** pest, chemotaxonomy, aphid, Taif Governate, CAP analysis, GC-MS headspace, closed-loop stripping

## Abstract

**Simple Summary:**

Aphids are small insects that damage plants by sucking plant phloem sap. Aphids cause severe losses to agriculture. In order to control aphids by environmentally friendly methods, it is necessary to identify them. Identification of aphids is difficult because there are ca. 5000 species which undergo different life stages and vary much in appearance (e.g., coloration). Here, we presented a novel method to determine aphid species using their chemical profiles. There are compounds (biomarkers) that are characteristic for a particular aphid species like a fingerprint. After grinding aphids, the released compounds were collected and the compound mixtures were separated by gas chromatography connected to a mass spectrometer to identify the individual compounds. The obtained compound profiles were subjected to the software CAP12.exe to reveal the biomarkers from the headspace profiles for each aphid species. It was possible to reliably differentiate aphid species using our approach. Compared to the established identification of aphids that uses characteristic DNA sequences from their genome, using the chemical biomarkers is much less laborious and faster. Moreover, it is possible with the chemical profile analysis to reveal other aspects of an aphid’s life history such as the food plant it fed on.

**Abstract:**

Aphids are a ubiquitous group of pests in agriculture that cause serious losses. For sustainable aphid identification, it is necessary to develop a precise and fast aphid identification tool. A new simple chemotaxonomy approach to rapidly identify aphids was implemented. The method was calibrated in comparison to the established phylogenetic analysis. For chemotaxonomic analysis, aphids were crushed, their headspace compounds were collected through closed-loop stripping (CLS) and analysed using gas chromatography—mass spectrometry (GC-MS). GC-MS data were then subjected to a discriminant analysis using CAP12.exe software, which identified key biomarkers that distinguish aphid species. A dichotomous key taking into account the presence and absence of a set of species-specific biomarkers was derived from the discriminant analysis which enabled rapid and reliable identification of aphid species. As the method overcomes the limits of morphological identification, it works with aphids at all life stages and in both genders. Thus, our method enables entomologists to assign aphids to growth stages and identify the life history of the investigated aphids, i.e., the food plant(s) they fed on. Our experiments clearly showed that the method could be used as a software to automatically identify aphids.

## 1. Introduction

Aphids (Hemiptera: Aphididae) are a ubiquitous group of insects, most commonly found in temperate regions [1]. Aphids are phloem suckers that can cause considerable damage to plants [2,3] and major losses in crop yield [4,5]. There are ca. 5100 aphid species that can be classified based on their morphology, genetics, host plant selection, and geography [6]. Aphids can be both mono- or polyphagous, colonising either individual genera/species or a wide variety of fruits, vegetables, and ornamental plants [7]. For example, *Aphis punicae* Passerini is a *Punica granatum* specialist, whereas *Aphis nerii* Fonscolombe, a *Nerium oleander* aphid, is reported to feed strictly on *Hypochoeris radicata* [8]. In contrast, *Aphis gossypii* Glover, *Aphis illinoisensis* Shimer, and *Aphis craccivora* Koch have a wide host range [9,10,11].

Rapid and accurate aphid identification is extremely important in entomological research, aphid control and aphid resource utilization [12]. Thus, sustainable methods to control aphids are needed to prevent losses in crop yield. An in-depth understanding of aphid ecology, associated insects, host plants, predators, and the role of microbial symbionts can lead to increased sustainable management practices in agriculture [13,14,15,16,17,18,19].

For this, it is crucial to identify the aphids. It can be very challenging to identify aphid species by morphological parameters, for example, because aphids undergo different growth stages. Even with excellent high-resolution picture databases such as Aphid Species File (http://Aphid.SpeciesFile.org/, accessed on 4 October 2020) [20] and AphID (http://aphid.aphidnet.org/index.php/, accessed on 31 March 2022) [21], the identification is not straightforward and limited to one sex and growth stage.

Therefore, other strategies to identify aphids are searched for. Clearly, phylogenetic analysis is a reliable strategy [22]. However, it is time consuming and fairly costly.

Perera, Vargas [23], and Thorpe, Cock [24] used protein profiles for aphid determination. Moreover, Chen, Bai [25] made use of the hydrocarbon pattern of *Acyrthosiphon pisum* for intraspecific delimitation. Also, IR patterns of aphids have been analysed using principal component analysis allowing differentiation of 12 aphid species [26]. Associated symbionts have been used to identify aphids, but this strategy is limited due to its dependence on the growth stage [27].

In general, the metabolic profiles of organisms can be regarded as their chemical fingerprints that may allow unambiguous differentiation between species. Such methodology is in particular well established for the determination of microorganisms based on their fatty acid methyl ester profiles after hydrolysis of their lipids [28,29,30]. There is even a software tool available relying on the fatty acid methyl ester pattern analysis for microbial identification http://midi-inc.com/, accessed on 2 February 2021 [31]. Alternatively, protein profiles are used in the Biotyper software developed by Bruker to quickly identify microorganisms [32]. With the increasing amount of data, there is a trend to establish valuable specific databases, e.g., pherobase (https://www.pherobase.com/, accessed on 26 May 2022) [33], mVOC [34], GNPS [35], or MACE [36] that simplify finding relevant information, for example, about pheromones or the biological function of volatile compounds.

However, in many cases, these databases are limited to date because they only list data for quick comparisons or identifications under narrow parameters. Therefore, there is an accelerated need to apply sophisticated and accurate metabolomic dataset in identification processes. For this, software tools have been established to support metabolomics or proteomics [37,38,39,40,41].

Here, we evaluated whether biomarkers in the headspace profiles of crushed aphids can be identified to allow straightforward, reliable species identification. This methodology could in the future both contribute to overcome limitations in aphid species identification for entomologists and constitute the basis to develop a software tool for fast and simple species identification for pest management.

## 2. Materials and Methods

Collection of aphids. Aphids were collected from different sites in Taif Governorate (21°16′0″ N, 40°25′0″ E), Saudi Arabia. Aphids were collected from flowers, leaves, or stems of different host plants (Table S1) between November 2021 and September 2022. The aphid samples were divided into two parts: the first part was kept in a 99% ethanol except green apple aphids which were washed with chloroform, ethanol, and hexane (1:1:1) to remove the waxy material secreted from this aphid; they were kept in a 99% ethanol and stored at −20 °C until use for molecular identification. The second part of samples was immediately used for headspace collection and GC-MS analyses (see below).

Headspace collection upon cell damage of aphids. Aphids (75–100 mg, around 10 to 20 individuals) were crushed in a 6 mL porcelain mortar with liquid nitrogen (3–6 replicates). The damaged aphid tissues were transferred into 10 mL vials with a wide opening (20 mm). The vials were tightly closed with a septum lid. The headspace of the vial was collected onto charcoal filters (3 mg) by closed-loop stripping (CLS), shuffling the headspace through the charcoal filters with a pump (model no. DC6/18 F; Fürgut GmbH, Jena, Germany) for 30 min [42,43]. Charcoal traps were eluted using 3 × 20 µL of ethyl acetate. The samples were analysed by GC-MS or stored at −80 °C until analysis. The charcoal filters were cleaned before use by rinsing with methanol (1.5 mL), followed by ethyl acetate (1.5 mL) and drying them at 80 °C for 2 h.

Some aphid species were covered with wax layers, for example, aphids collected from green apple tree. These aphids were rinsed twice as fast as possible with a mixture of 3 to 5 mL of ethanol, chloroform, and hexane (1:1:1) to remove the wax layers and lipids which are secreted by the aphids. Because of this rinsing step, ca. 300 mg of crushed aphids was needed for the headspace analysis to overcome headspace volatile losses.

To exclude any potential interferences because of mixing of different aphids in a sample during the collection, the method was performed using an individual of each aphid species as follows: one aphid was crushed in a glass vial under liquid nitrogen, and CLS was directly conducted for 45 min.

Data analysis and identification of headspace compounds by GC-MS. Samples were analysed using a 580 Clarus gas chromatograph (GC) coupled to a 560S Clarus mass spectrometer (MS) (Agilent Technologies, Santa Clara, CA, USA). The GC was equipped with a non-polar Elite-5MS capillary column (30 m × 0.25 μm, 0.25 μm film thickness, Agilent Technologies, Santa Clara, CA, USA) using helium as a carrier gas at a constant flow rate of 1 mL/min. The GC-MS conditions were the following: the injection pre-dwell time was set to 0.1 min (hot needle injection). The injector temperature was set to 220 °C. A total of 1 µL was injected into the GC port using spitless mode. The temperature was increased to 130 °C at 2.8 °C/min, followed by an increase to 180 °C at 3 °C/min, where it was ramped to a final temperature of 250 °C at 6 °C/min. The MS was operated at 70 eV, trap current emission: 100 µA, source temperature: 150 °C, transfer line temperature: 280 °C. Scanning was performed from m/z 40 to 450 amu. Data acquisition and analysis were carried out using Turbomass software (SQ8 V6.1.2, Perkin Elmer, Waltham, MA, USA). The compounds were identified by analysis of their mass spectra and comparison with the NIST14 database (Gaithersburg, MD, USA). Selected compounds were verified by co-injection of authentic standards.

Data pre-processing and statistical analysis. The chromatograms of all technical replicates (*n* ≥ 3) were pre-processed (see Section S2.3 in SI) and the means of replicates were used for canonical analysis of principle coordinates using CAP12.exe (version 12, University of Auckland, Auckland, New Zealand). http://www.stat.auckland.ac.nz/~mja, accessed on 18 June 2020 [44]. Scatter plots to visualize the distribution of the samples and vector plots in order to collect biomarkers for discriminating aphid species were drawn using SigmaPlot (version 11.0, Systat software, Bayshore, NY, USA, see Section S2.3 in SI). All analysed aphid samples were subjected to phylogenetic identification to verify the efficiency of the chemotaxonomy approach.

Molecular identification of aphids. DNA was extracted from samples of the isolated aphids based on CAP analyses (see Sections S2.2 and S2.3 in SI). Five to seven individuals from each preserved species were subjected to the QIAquick protocol for DNA isolation, rinsed with phosphate buffered saline (10 mM Na_3_PO_4_, 150 mM NaCl, pH 7.4) to remove ethanol that aphids were stored in. Subsequently, a pool of each species was homogenised, and DNA was isolated using the QIAamp^®^ DNA Mini Kit (QIAGEN, Düsseldorf, Germany) according to the manufacturer’s instructions. DNA concentrations and purity were determined using a NanoDrop spectrophotometer (Thermofisher Scientific, Wilmington, DE, USA). Extracted DNA (21–45 ng/mL) from each species was used as a template to amplify the COI gene using primers LCO1490-1-F (5′-GGTCAACAAATCATAAAGATATTGG-3′) and HCO2198-R (5′-TAAACTTCAGGGTGACCAAAAAATCA-3′) (Macrogen, Seoul, Republic of Korea) [45]. Amplification of COI was conducted in a thermocycler (Applied Biosystems, Wilmington, DE, USA) using 5× FIREPol^®^ Master Mix Ready to Load (Solis BioDyne, Tartu, Estonia). The PCR conditions were as follows: pre-denaturation at 94 °C for 15 min, followed by 35 cycles of denaturation (95 °C for 45 s), annealing (55 °C for 45 s), extension (72 °C for 45 s), and a final extension at 72 °C for 10 min. The PCR products were then analysed using 1.5% agarose gels with a 100 bp DNA ladder (Solis Biodyne). DNA bands were purified from the gel using the QIAquick gel extraction kit (Qiagen, MD, Hilden, Germany) according to the manufacturer’s instructions.

## 3. Results and Discussion

### 3.1. Aphid Identification by Phylogenetic Analysis

In total, 112 samples of aphids from different plants and geographical locations in Taif Governorate were identified based on the cytochrome oxidase subunit I (COI) gene [46]: *Aphis punicae* P., *Aphis gossypii* G., *Aphis craccivora* K., *Aphis illinoisensis* S., *Aphis nerii* F., *Rhodobium porosum* S., *Macrosiphum rosae* L., *Eriosoma lanigerum* H., *Lipaphis pseudobrassicae* D., and *Hysteroneura setariae* T. The sequences of each species were submitted to GenBank, and their accession numbers are given in Table S1. The COI gene sequence was used to construct a phylogenetic tree of the collected aphids which revealed that 112 samples of aphids were classified into ten different identified aphid species (Figure S1, Section S2.1 in SI). The phylogenetic tree served to name the species identified by our chemotaxonomic approach.

### 3.2. Chemotaxonomic Approach to Differentiate Aphid Species

Aphid samples were crushed in liquid nitrogen immediately after their collection from the host plants (Table S1). The collected headspace compounds were analysed by GC-MS and 81 compounds were extracted (Table S2, Section S2.2 in SI). A total of 79 biomarkers out of 81 were identified and classified to seven classes: terpenes (not present in *E. lanigerum*); ketones (not present in *A. gossypii* and *M. rosae*); aldehydes (not present in *E. lanigerum*, *M. rosae*, and *A. gossypii*-Vinca); and esters, which occurred in all aphid profiles (not present in *M. rosae*, *L. pseudobrassicae*, *A. craccivora*, and *A. gossypii*-lemon, -vinca and -mint. Two alkanes were detected in *M. rosea* and up to 16 were detected in *A. punicae*. Alkenes were only detected in *E. lanigerum.* Benzenoids were observed in the headspace of all aphid species except that of *M. rosae* and *A. gossypii*-vinca. In addition, alcohols, isothiocyanates, amines, cycloamides, and unidentified compounds were present in the headspace profiles of all investigated aphids (Table S1).

**Figure 1:**
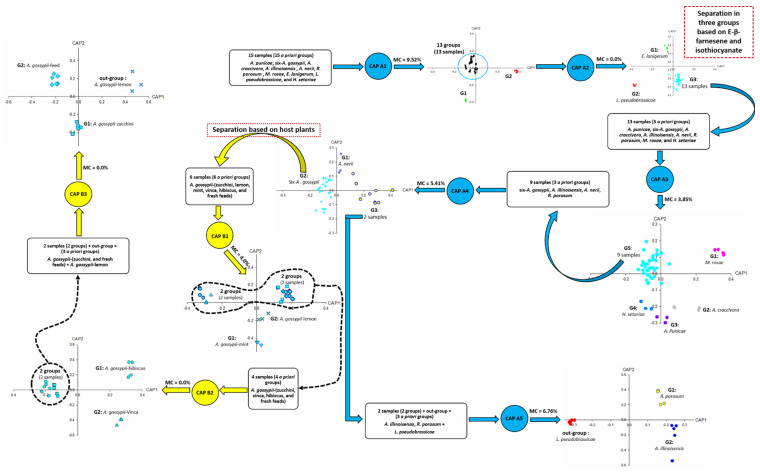
Headspace compounds released by aphids upon cell damage, analysed by CLS-GC/MS and subjected to CAP analysis. Blue circles and arrows indicate CAP analyses used for aphid species identification. Yellow circles and arrows indicate CAP analyses for host plant identification in case of polyphagous aphids-*A. gossypii*. CAP A1–A5 refers to Figure 2, Figure 3, Figure 4, Figure 5 and Figure 6, CAP B1–B3 refers to Figure 7, Figure 8 and Figure 9. A *priori* groups: three or more groups are suggested before processing CAP analyses based on host plants. Outgroup: it is a group added to perform CAP as the minimum number of a priori groups to run CAP is three. MC: misclassification. G: group.

The 81 compounds were subjected to multivariate analysis, i.e., canonical analysis of principle coordinates (CAP) (Figure 1) to identify biomarkers in the headspace that are characteristic for aphid species. We used CAP12.exe, a program for discriminant analysis on the basis of selected distance measure which is also seen as a data reduction technique [44]. CAP, however, is considered a multivariate descriptive method for analysing group partitioned data [47]. GC datasets of aphid species were separated by CAP based on the absence and existence of compounds or the abundance of compounds among headspace profiles. Each compound represents a vector. The longer vectors are the more significance vectors in aphid separation. To apply the data shown in Table S2 in the classification of aphid species as an alternative to phylogenetic identification (Figure S1), CAP was performed on the 81 metabolites found in the 10 aphid species (Table S1).

**Figure 2:**
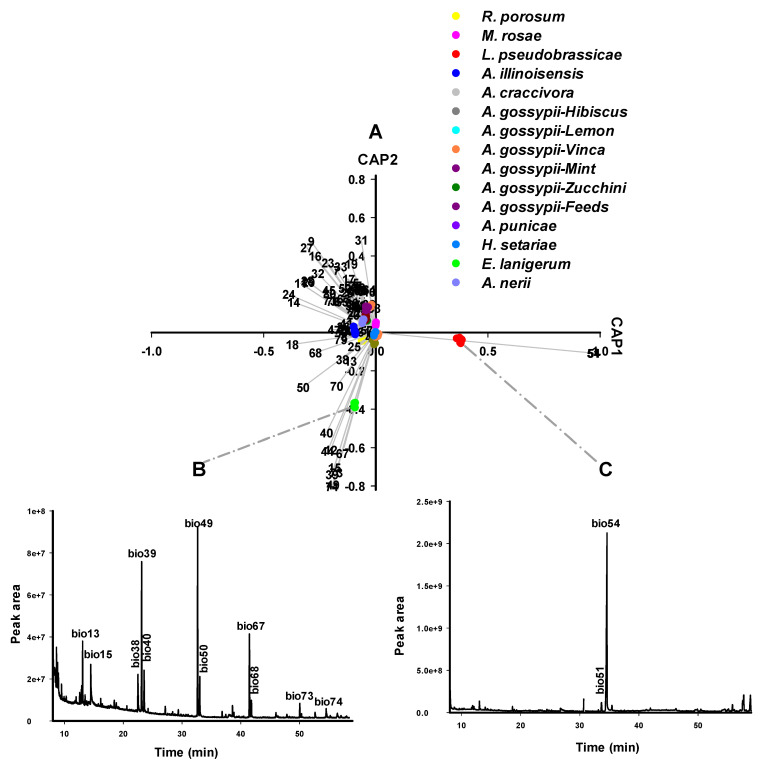
CAP analysis (CAP A1-first processing round) of the headspace compounds (*n* = 81) released by aphids upon cell damage and analysed by GC/MS (63 samples): (**A**) The first two canonical axes of the canonical analysis of principle coordinates (CAP) analysis are plotted and CAP analysis demonstrates the separation of the samples based on the presence and absence of headspace compounds. Scaled vectors of the headspace compounds (ID numbers) were significant (|r| > 0.2) for the separation of the groups, miss-classification error = 9.52%. The numbers refer to the headspace compounds in Table S2. (**B**) Chromatogram of *E. lanigerum* upon cell damage. (**C**) Chromatogram of *L. pseudobrassicae* upon cell damage. Values were calculated from three replicates.

**Figure 3:**
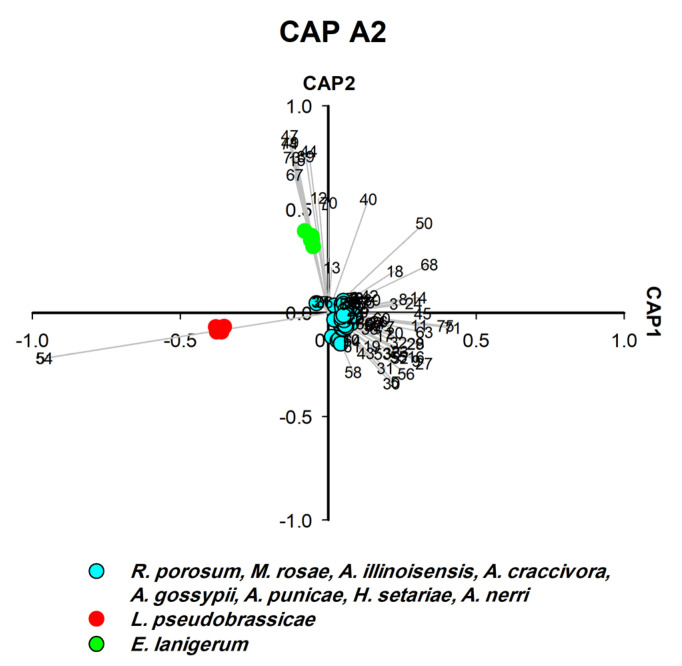
CAP analysis (CAP A2-second processing round) of the headspace compounds (*n* = 81) released by aphids upon cell damage and analysed by GC/MS (63 samples): the first two canonical axes of the canonical analysis of principle coordinates (CAP) analysis are plotted and CAP analysis demonstrates the separation based on the presence and absence of (1) (E)-β-farnesene, (2) isothiocyanates, (3) other headspace compounds. Scaled vectors of the headspace compounds (ID numbers) were significant ($\sqrt{X^{2}+Y^{2}}$ > 0.65) for the separation of the groups, miss-classification error = 0.0. The numbers refer to the headspace compounds in Table S2.

**Figure 4:**
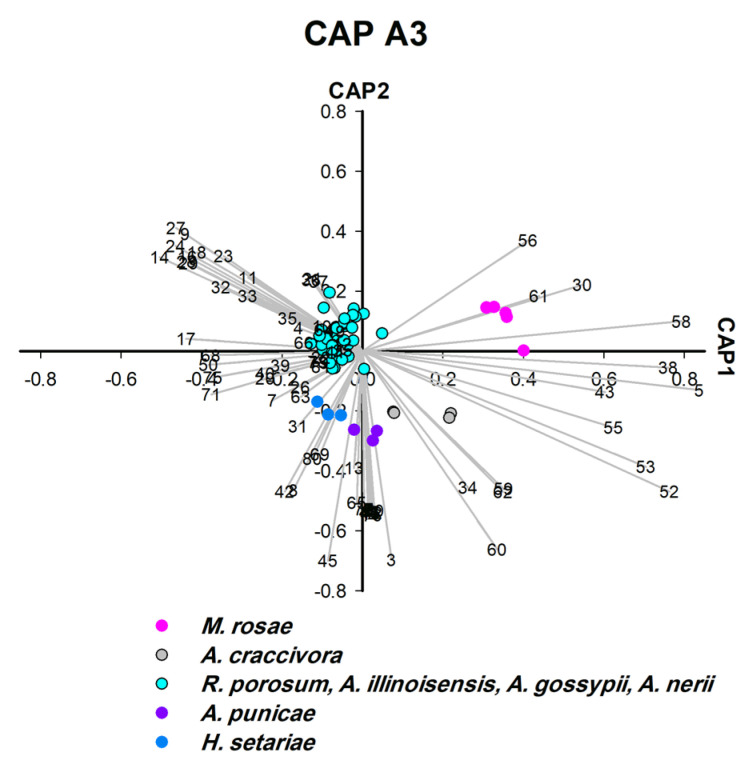
CAP analysis (CAP A3-third processing round) of the headspace compounds (*n* = 74) released by aphids upon cell damage and analysed by GC/MS (52 samples): the first two canonical axes of the canonical analysis of principle coordinates (CAP) analysis are plotted and CAP analysis demonstrates the separation based on the present and absence of headspace compounds; Scaled vectors of the headspace compounds (ID numbers) were significant ($\sqrt{X^{2}+Y^{2}}$ > 0.3) for the separation of the groups, miss-classification error = 3.85%. The numbers refer to the headspace compounds in Table S2.

**Figure 5:**
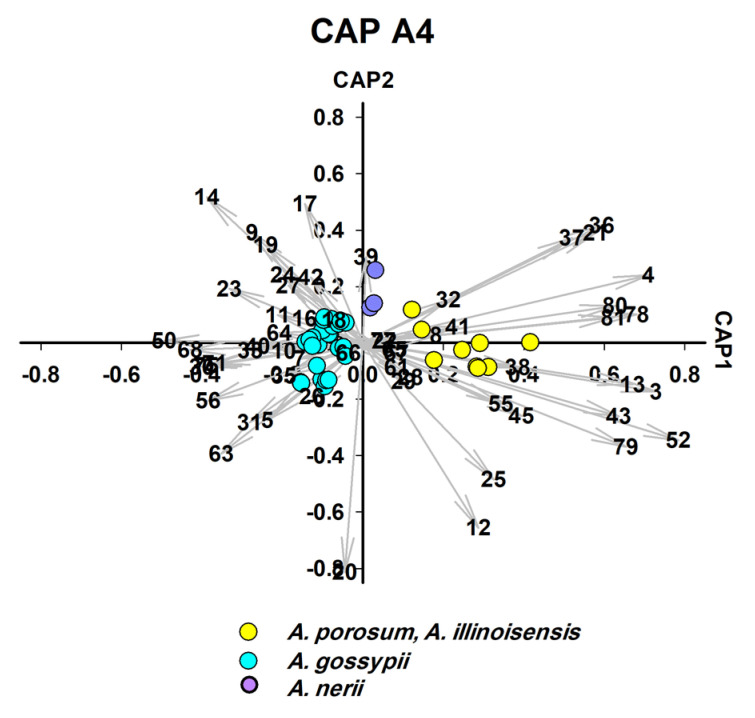
CAP analysis (CAP A4-fourth processing round) of the headspace compounds (*n* = 57) released by aphids upon cell damage and analysed by GC/MS (37 samples): the first two canonical axes of the canonical analysis of principle coordinates (CAP) analysis are plotted and CAP analysis demonstrates the separation based on the present and absence of headspace compounds. Scaled vectors of the headspace compounds (ID numbers) were significant ($\sqrt{X^{2}+Y^{2}}$ > 0.3) for the separation of the groups, miss-classification error = 5.41%. The numbers refer to the headspace compounds in Table S2.

**Figure 6:**
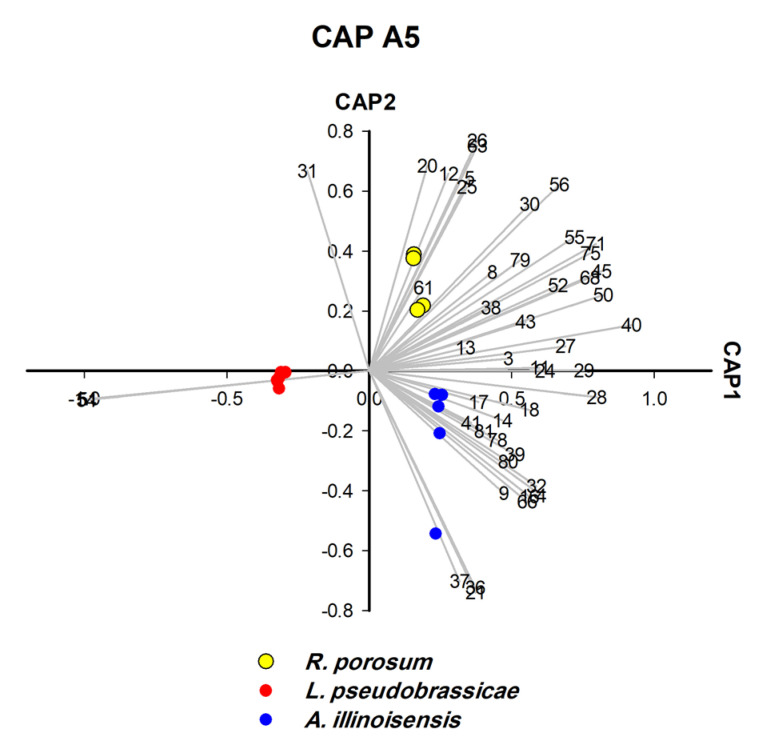
CAP analysis (CAP A5-fifth processing round) of the headspace compounds (*n* = 47) released by aphids upon cell damage of 15 samples and measured by GC/MS: the first two canonical axes of the canonical analysis of principle coordinates (CAP) analysis are plotted and CAP analysis demonstrates the separation based on the present and absence of headspace compounds. Scaled vectors of the headspace compounds (ID numbers) were significant ($\sqrt{X^{2}+Y^{2}}$ > 0.65) for the separation of the groups, miss-classification error = 6.67%. The numbers refer to the headspace compounds in Table S2.

**Figure 7:**
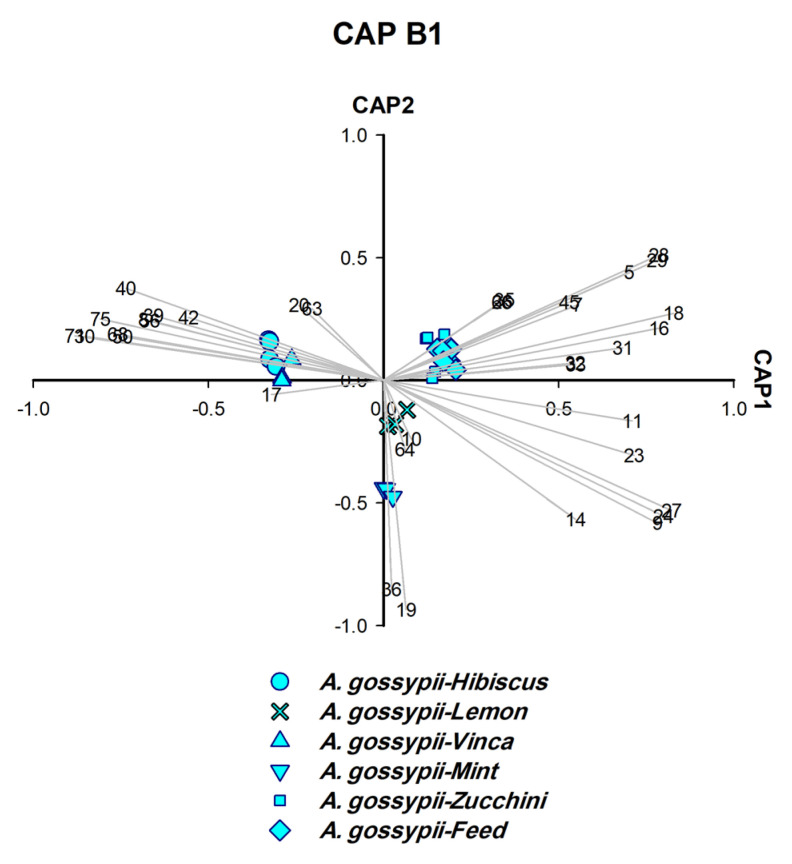
CAP analysis of the headspace compounds (*n* = 36) released by aphids upon cell damage of 25 samples of *A. gossypii* analysed by GC/MS: the first two canonical axes of the canonical analysis of principle coordinates (CAP) analysis are plotted and CAP analysis demonstrates the separation based on the presence and absence of headspace compounds. Scaled vectors of the headspace compounds (ID numbers) were significant ($\sqrt{X^{2}+Y^{2}}$ > 0.25) for the separation of the groups, miss-classification error = 4.0%. The numbers refer to the headspace compounds in Table S2.

**Figure 8:**
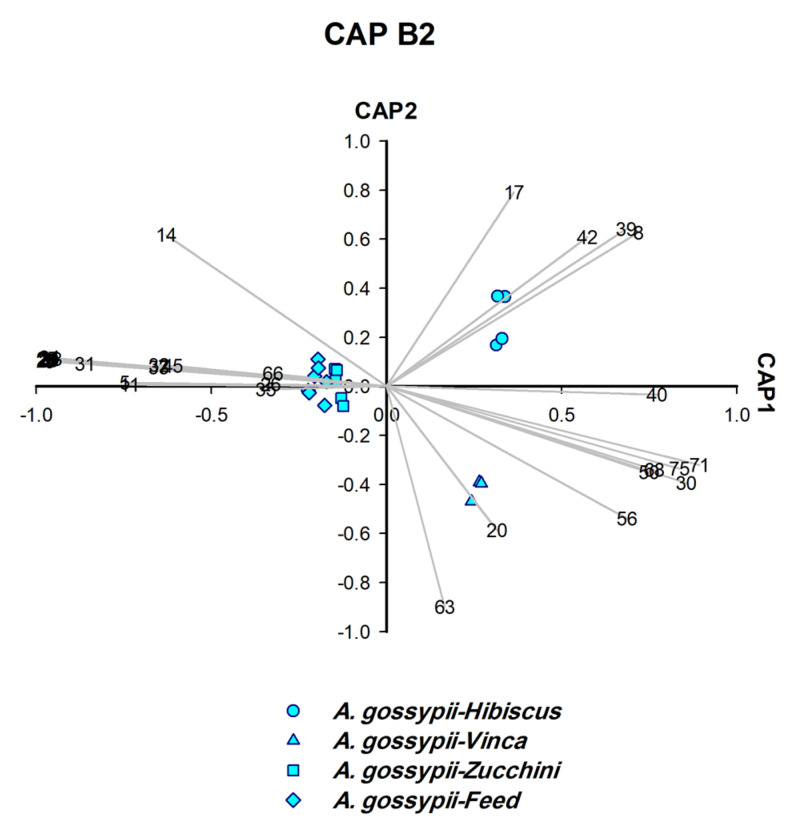
CAP analysis of the headspace compounds (*n* = 32) released by aphids upon cell damage of 19 samples of *A. gossypii* analysed by GC/MS: the first two canonical axes of the canonical analysis of principle coordinates (CAP) analysis are plotted. CAP analysis demonstrates the separation based on the presence and absence of compounds in the headspace. Scaled vectors of the headspace compounds (ID numbers) were significant ($\sqrt{X^{2}+Y^{2}}$ > 0.66) for the separation of the groups, miss-classification error = 0.0%. The numbers refer to the headspace compounds in Table S2.

**Figure 9:**
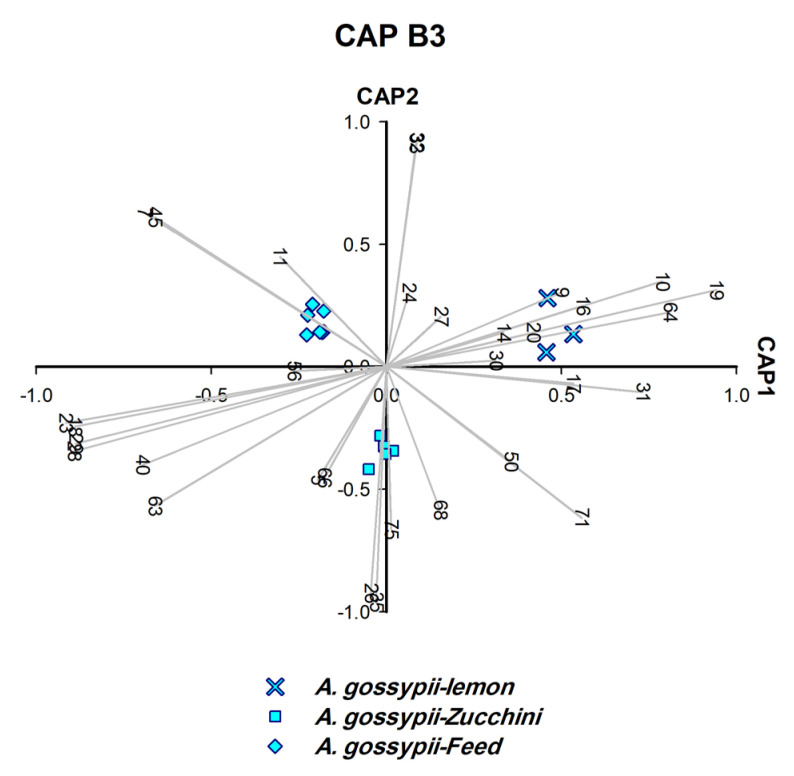
CAP analysis of the headspace compounds (*n* = 32) released by aphids upon cell damage of 15 samples of *A. gossypii* analysed by GC/MS. The first two canonical axes of the canonical analysis of principle coordinates (CAP) analysis are plotted and CAP analysis demonstrates the separation based on the presence and absence of headspace compounds. Scaled vectors of the headspace compounds (ID numbers) were significant ($\sqrt{X^{2}+Y^{2}}$ > 0.9) for the separation of the groups, miss-classification error = 0.0%. The numbers refer to the headspace compounds in Table S2.

For polyphagous aphids such as *A. gossypii*, we also used the CAP analysis to identify the plant on which the aphids fed. Obviously, it was possible to find characteristic markers to accurately identify the host plant of these aphids. Figure 1 is an illustration of how the strategy was implemented forc ollected aphids. Two different sets of CAP analysis were performed, the first set, CAP A1–A5 (Figure 2, Figure 3, Figure 4, Figure 5 and Figure 6), was used to identify aphid species and the second set, CAP B1–B3 (Figure 7, Figure 8 and Figure 9), was performed to determine the host plants of polyphagous *A. gossypii*.

### 3.3. The Aphid Alarm Pheromone (E)-β-Farnesene (EβF) as Selection Criterion (CAP A1)

The first round of CAP-A1 analysis separated 15 aphid samples into three main groups: *L. pseudobrassicae*, *E. lanigerum*, and a group containing the remaining 13 samples (comprising eight aphid species) (Figure 2). Under the selected parameters in CAP analysis, 13 samples were classified in a single group regardless of species variety, host plants, or geographical location, except for *L. pseudobrassicae*, and *E. lanigerum*. The principal factor contributing to this classification was the aphid alarm pheromone EβF [48], which was not detected in the headspace of the latter two aphid species. The *L. pseudobrassicae* headspace was characterised by trans-raphasatin and cis-raphasatin, while *E. lanigerum* headspace was distinguished by pentadecane, 1-tetradecanol, docosane, decane, 8-heptadecene, and 9-eicosene. The chemical differentiation corresponded well to the apparent molecular cluster in the phylogenetic tree (Figure S1). EβF cannot be a criterion for the chemotaxonomy of most aphid species due to its lack of selectivity as it is widespread among many aphid species. Thus, further rounds of CAP analysis are required for identification of these aphid species

### 3.4. Hydrocarbon Profiles as Biomarkers for Aphid Species Differentiation (CAP A2–5)

CLS-GC-MS analysis revealed that a single hydrocarbon was recorded for *L. pseudobrassicae*, and 16 hydrocarbons were recorded for *A. punicae* (Table S2). In general, among all aphid species, the most frequently detected alkanes were 4,5-dimethylnonane (n = 14), tetradecane (n = 13), 2,6,10,15-tetramethylheptadecane (n = 13), heptacosane (n = 12), and octacosane (n = 12) (Table S2). Linear hydrocarbons (C10–C44) contributed significantly to the separation of four species (Figure 4, Figure 5 and Figure 7) and methyl-branched alkenes (Me-Alk) (C11–C21) separated two species (Figure 5). Both linear alkanes and methyl branched alkanes act as significant biomarkers for two species (Figure 5 and Figure 6), whereas no hydrocarbon was characteristic for *L. pseudobrassicae* and *A. nerii* (Figure 4 and Figure 6). It was apparent that hydrocarbons that were detected in the headspace profile (Table S2) likely originate from symbionts associated with the aphids [49], plants [50] and aphid cuticular hydrocarbons [51]. Saturated alkanes (C13-C28) were also reported in previous studies as aphid cuticular hydrocarbons) from *A. craccivora* [52], *Brevicoryne brassicae*, *Hyalopterus pruni* [53], and *Aphis fabae* [54]). Decane (detected in *E. lanigerum* and *A. punicae*), 9-eicosene, and 8-heptadecene (detected in *E. lanigerum*) were detected in the headspace hydrocarbons (Figure 2, Table S2) and were also found by Muñoz, Argandoña [55], Ahmed, Agarwal [56], by Pokharel, Zhong [57] in aphid-infested plants, and by Ali, Morgan [58] in Leptothorax ants. Among Me-Alk, dimethyl alkanes were detected in the headspace of crushed aphids (Figure 2, Table S2), i.e., 3,8-dimethyl undecane, 4,7-dimethyl undecane, 4,5-dimethyl, nonane, and 4,6-dimethyl dodecane. Despite the significant role of hydrocarbons in aphid identification which is investigated in this study, hydrocarbons play an important role in the determination of aphid age [59]. To conclude, alkanes and methyl branched alkanesare important for identifying aphid species and are rather useful for determining the history of infestation (see SI, Sections S3.5 and S3.6).

### 3.5. Isothiocyanates Characterise Aphid Species Feeding on Brassicaceae (CAP A2)

Glucosinolate-derived volatile breakdown products, such as trans- and cis-raphasatine, were identified as significant biomarkers by CAP analysis (Figure 2, Table S2) of aphids that feed on glucosinolate-producing *Brassicaceae*. Glucosinolates act as precursors for activated defense in *Brassicaceae*. Toxins, such as volatile isothiocyanates, nitriles, epithionitriles, thiocyanates or oxazolidines, are released upon cell damage when glucosinolates mix with myrosinase that are stored in different compartments [60]. The ploem-sucking aphids overcame this plant defense because they do not destroy the cell structure and thus compartmentalization of glucosinolates and myrosinase. Moreover, aphids sequester glucosinolates and use them for their own defense [61,62,63]. We collected *L. pseudobrassicae* from *Raphanus sativum* (radish) that sequesters the glucosinolates from the plant source and upon damage (by homogenization) converts the glucosinolates using its myrosinase to trans- and cis-raphasatine. Thus, host-plant-derived volatiles can constitute valuable biomarkers for aphid identification.

Each aphid species has its specific biomarker pattern like a fingerprint. Thus, all aphids feeding on *Brassicaceae* share the raphasatine in their headspace profile [64,65], but differ in other biomarkers. Therefore, we recommend applying the chemotaxonomic approach to *Brevicoryne brassicae* and *Lipaphis erysimi* to find out the significant difference between the headspace profiles of *Brassicaceae*-feeding aphids

### 3.6. Terpene Profiles of Aphid Species Feeding on Taif Rose (CAP A3/A5)

Molecular identification revealed strong clustering of *R. porosum* and *M. rosae* (posterior probability = 90%) (Figure S1) which are feeding on Taif rose (*Rosa damascene*, Rosaceae). The two aphid species were successfully separated by third and fifth rounds of CAP analysis (Figure 4 and Figure 6). In terms of significant biomarkers, hexadecane and benzenoid derivative (bnz-26) were detected in *R. porosum*-Taif rose, whereas 4, 5-dimethyl nonane, azulene, and UK-58 were detected in *M. rosae*-Taif rose. The two alkanes (hexadecane and 4, 5-dimethyl nonane) are the chemotaxonomic biomarkers of *R. porosum* and *M. rosae*, respectively (Figure 4 and Figure 6). However, trans-α-bergamotene and cyclofenchene were detected in the headspace of both aphid species (Figure 4 and Figure 6, Table S2), most likely related to the same host plant—Taif rose. Two additional unidentified biomarkers (UK-12 and UK-25) were found in the headspace of *R. porosum* (with retention times of 12.97 min and 16.54 min, respectively) which may be classified as terpenes or benzenoids according to the retention time range of each substance class presented in Table S2.

The headspace profile of *M. rosae*—Taif rose located in Alhada (high mountain area; N21.3388736, E40.3242934) emitted less headspace compounds (10 biomarkers, with the virtual disappearance of benzenoids, esters, and aldehydes; Table S2) than *R. porosum*-Taif rose located in the lower altitudes of Alhawia (desert lowland; N21.428299, E40.473267) in a greenhouse (32 biomarkers; Table S2). The observed influence of altitude and sun intensity on the quantity and quality of volatile bouquet emitted by aromatic plants, i.e., *R. damascene* is in accordance with the results of Gouinguené and Turlings [66], Dudareva, Klempien [67], Rusanov, Kovacheva [68], but in contradiction with those of Du et al. [69].

### 3.7. Identification of Host Plants of Aphid from Aphid Headspace Profile (CAP B1–B3)

*A. gossypii* was separated as individual species in the fourth round of CAP analysis (Figure 5), although samples were collected from six different host plants. The chemotaxonomic identification of all six isolates as one species characterised by same six significant biomarkers including EβF (Figure 5) was supported by the molecular identification which verified that the six isolates are *A. gossypii* (Figure 5 and Figure S1).

To determine the plant on which *A. gossypii* were feeding, another set of CAP analyses (CAP B1–B3) was carried out on the six samples obtained (Figure 7, Figure 8 and Figure 9). CAP analysis played a significant role in identifying the host plants on which *A. gossypii* fed (*n* = 6). The biomarkers generated by CAP B1–B3 (Figure 7, Figure 8 and Figure 9) belong to two categories: (A) monoterpenes that represented the constitutive plant defenses (*o*-cymene, *L*-menthone, *β*-thujene, and *D*-limonene), and (B) phenylpropanoid pathway products (benzaldehyde and methyl salicylate (MeSA)).

Three host plants of *A. gossypii* were determined as follows: *o*-cymene and *L*-menthone identified mint, *β*-thujene characterised lemon (Figure 7), and *D*-limonene distinguished vinca (Figure 8). The remaining three host plants were characterised by biomarkers other than monoterpenes, i.e., zucchini (derivative: Bnz26), hibiscus (MeSA, benzaldehyde and caprolactam), and fresh feed (tridecane and benzenoid derivative: Bnz7) (Figure 8 and Figure 9).

### 3.8. Generation of the Dichotomous Key from CAP Analysis

**Figure 10:**
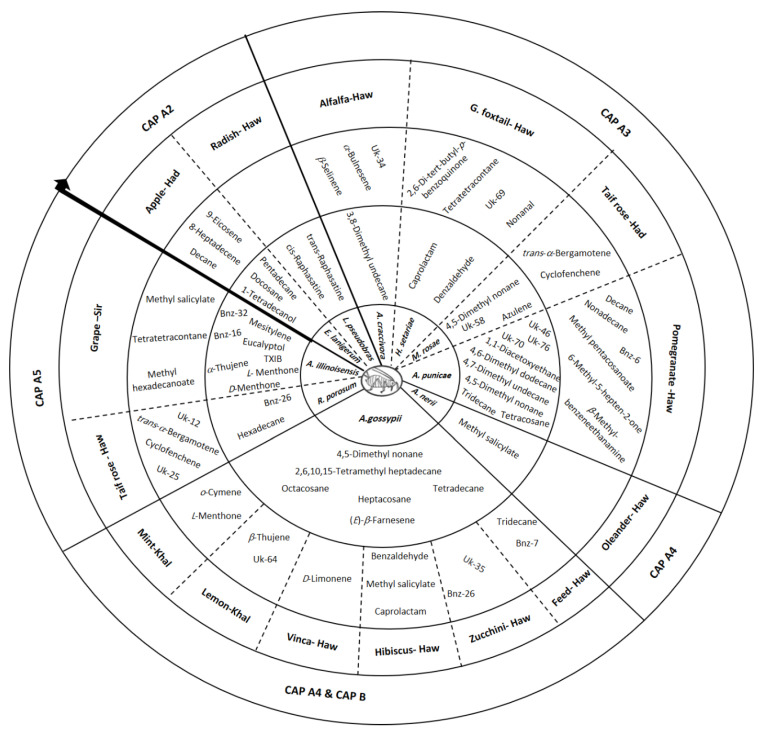
Dichotomous key for aphid identification consists of EB (internal circle) and SEB (external circle). CAP A2–5 refers to Figure 3, Figure 4, Figure 5 and Figure 6 and CAP B refers to Figure 7, Figure 8 and Figure 9. Had, Haw, Khal: Alhada, Alhawia, and Alkhaldia are the locations from where aphids were collected. Uk: unknown biomarker. Bnz: benzene derivatives. TXIB: 2,2,4-trimethylpentane-1,3-diyl bis(2-methylpropanoate).

Species-specific biomarkers were found for all investigated aphid samples (Figure 10). Thus, the current methodology can be used to determine aphid species. Our chemotaxonomic approach is less laborious and four times faster than the phylogenetic analysis. The whole procedure requires a maximum of 2.5 h, while PCR and sequencing needs at least several hours (ca ≈ 10 h) [70]. The method can also work with a single aphid if one optimizes the CLS-GC-MS method using long enough headspace collection (45 min) and sensitive splitless injection. Our experiments confirmed that the results obtained with 75 to 100 mg of aphids or only one aphid were the same (Figures S4 and S5, see Section S3.9, SI). Moreover, the method works for aphids at different life stages, even with nymphs, and thus overcomes limitations the morphological identification has.

The biomarkers thus obtained by CAP analysis were classified into two groups: essential and semi-essential biomarkers (EBs and SEBs, respectively) which were used to construct a dichotomous key (Figure 10). EBs and SEBs classified aphid species (Figure 2, Figure 3, Figure 4, Figure 5 and Figure 6) as well as identified their host plants (Figure 7, Figure 8 and Figure 9). After the intervention of all rounds of CAP analysis (CAP A2–5), GC-MS data were reduced and displayed in a dichotomous key (Figure 10).

EBs are the longest vectors (0.49 ≤ $\sqrt{X^{2}+Y^{2}}$ ≤ 0.97) where (X, Y) are the vector coordinates on X and Y axes in the CAP analysis. The longest vectors are mainly alkanes and aldehydes that make up the inner circle of the dichotomous key (Figure 10). On the other hand, the shortest vectors with lower correlation coefficients were considered to be SEBs (0.29 ≤ $\sqrt{X^{2}+Y^{2}}$ < 0.49). These short vectors are primarily alkenes, terpenes and esters that form the outer circle in the dichotomic key (Figure 10). Both EBs and SEBs represent a distinguished aphid pattern. It is worth mentioning that the values of $\sqrt{X^{2}+Y^{2}}$ were chosen depending on the shape and length of vectors according to each CAP analysis. For more details about the developed strategy, in particular the CAP analysis, see Section S2.3 in SI.

The chemotaxonomy approach for identifying aphid species can be summarized as follows: The headspace compounds presented in the dichotomous key indicate that alkanes account for most EBs and contribute to the identification of most aphid species (i.e., *E. lanigerum*, *A. craccivora*, *M. rosae*, *A. punicae*, *A. gossypii* and *R. porosum*). While *L. pseudobrassicae* was identified by isothiocyanate, other aphid species were identified differently due to the disappearance of alkanes in EBs (Figure 10). For instance, it can be clearly seen that aldehydes, esters, benzenoids, and terpenes represent EBs of *H. setariae*, *A. nerii*, and *A. illinoisensis* in the dichotomous key (Figure 10), and thus they were used for their identification. Although some headspace compounds like alkanes, alkenes, esters, ketones, aldehydes, and terpenes were detected as SEBs, caprolactam, MeSA, and benzaldehyde play a dual role as EBs and SEBs (Figure 10).

It is obvious that the dichotomous key relies mainly on alkanes and volatile organic compounds and it is therefore very important to avoid volatile losses during the extraction process of the headspace compounds (see headspace collection in Section 2, SI, Section S3.8, Figure S3).

### 3.9. Potential Production of Alkanes by Aphid Species in Taif Governorate (Saudi Arabia)

**Figure 11:**
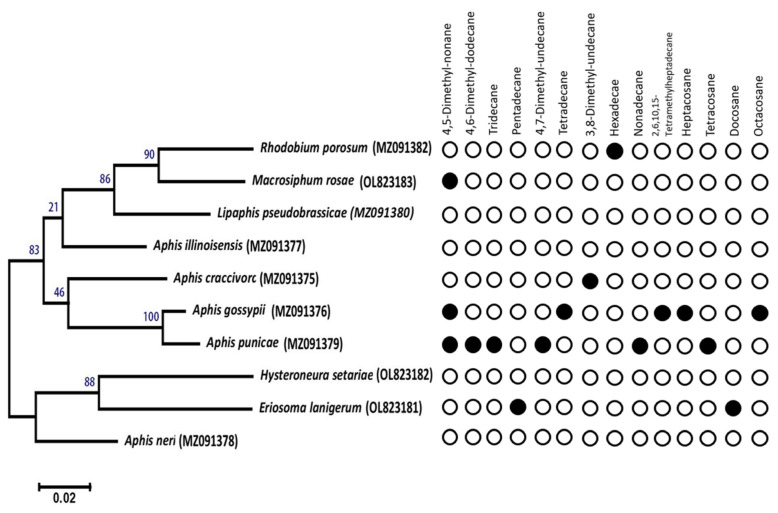
Phylogenetic tree and chemotaxonomic approach of alkanes in the aphid headspace. Filled circles indicate alkane is an essential biomarker based on CAP analysis. Open symbols indicate alkane is not an essential biomarker in the headspace of the respective aphid sample. GenBank accession numbers are given in brackets for reference taxa (see complete list in Table S1). Scale bar = 0.02 base substitutions per nucleotide.

Based on the dichotomous key (Figure 10), alkanes were the most prominent EBs among the collected aphid species from Taif Governorate. Six out of ten aphid species were identified by alkanes and Me-Alk. The aphids which were collected were subjected to the CLS-GC/MS-CAP approach. However, gene-sequencing-based classifications of aphids are so far promising for proper species identification (Figure S1). Therefore, we correlated the phylogenetic tree with alkanes (Figure 11) to exhibit the chemotaxonomic significance of the perspective biosynthetic pathway. However, further studies must be conducted to analyse whether the amount and pattern of alkanes vary throughout the different aphid species, growth stages, collection sites. Indeed, such information is of particular interest in entomological research.

### 3.10. Rapid Diagnosis of Aphid Species by GC-MS and CAP Analysis

Unknown green aphids were collected from sow-thistle (*Asteraceae*) in February 2022, Taif Governorate. Five farmers and five scientists were asked to determine the aphid species. Based on a questionnaire shown in Table 1, both farmers and scientists could not identify the unknown aphid. Identification using morphological criteria of aphid (AphID; http://aphid.aphidnet.org/index.php, accessed on 31 March 2022) [21] was attempted but the collected unknown aphids were nymphs and thus could not be identified.

**Figure 12:**
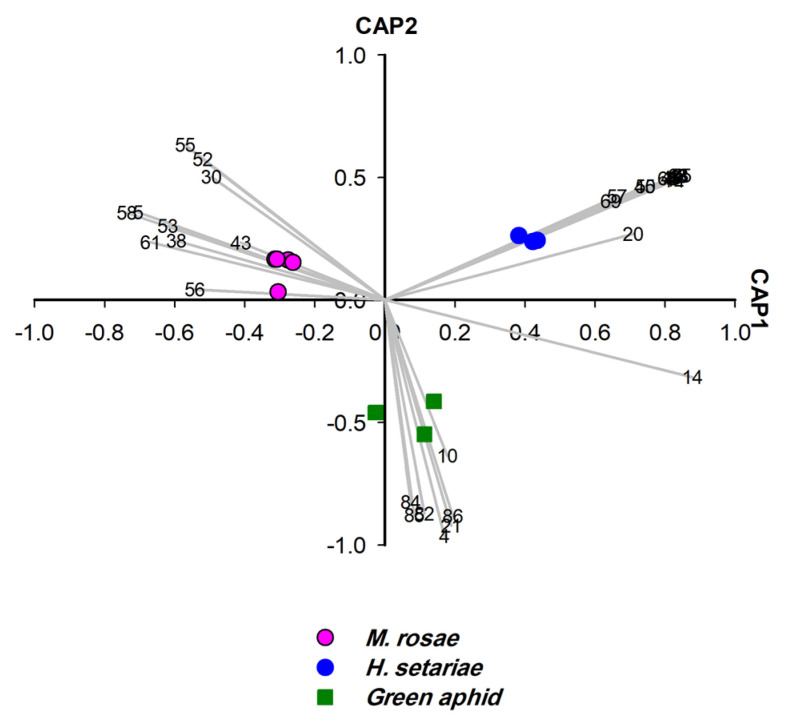
CAP analysis of the headspace compounds (*n* = 43) released by aphids upon cell damage of 14 samples analysed by GCMS: the first two canonical axes of the canonical analysis of principle coordinates (CAP) are plotted and CAP analysis demonstrates the separation based on the presence and absence of headspace compounds. Scaled vectors of the headspace compounds (ID numbers) were significant ($\sqrt{X^{2}+Y^{2}}$ > 0.46) for the separation of the groups, miss-classification error = 0.0%. The numbers (1–81) refer to the headspace compounds in Table S2 and (82–96) refer to the headspace compounds in Table 2.

The unknown aphid species was subjected to CLS-GC-MS analysis and chemotaxonomic approach (three replicates). The unknown green aphid clearly differed from *M. rosae* (100%). In total, 11 headspace compounds were identified for the unknown green aphid (Table S3, SI Section S3.7). Based on CAP analysis (Figure 12), six terpenes were dominant and constituted the EBs, whereas β-thujene represented a SEB and no alkane was detected in EBs or SEBs (Table 2).

The headspace of the crushed unknown green aphid contained monoterpenes in a large amount. This unique composition of monoterpenes differentiated it from *M. rosae*. Several studies established *α*-pinene as the main alarm pheromone component of aphids, whereas EβF occurred only in low amounts [71,72,73,74]. It was reported by Song, Qin [74] that α-pinene content is higher during the second instar (>81%) compared to that during the other growth stages of aphids, whereas EβF was higher in the third instar compared to that during the other growth stages of aphids. Thus, the chemotaxonomic approach indicated that the unknown green aphids were likely collected in their second instar of growth stage, and this has been proven by the morphological identification under the microscope.

To confirm the result of the chemotaxonomic approach, the unknown green aphid subjected to molecular identification. The nucleotide sequence of the COI gene revealed that the analysed unknown green aphid was *Hyperomyzus carduellinus*, which is different from *M. rosae*. Both aphid species belong to the same family of Aphididae.

## 4. Conclusions and Future Perspectives

Rapid and reliable identification of aphids is very important for entomological research and aphid management [75]. To identify an unknown aphid using traditional DNA barcoding [70] and morphological methods (http://aphid.aphidnet.org/index.php/, accessed on 31 March 2022) [20] is time consuming and requires expert opinion.

Although recently principal component analysis and canonical correlation analysis were implemented in morphological analysis (TPSDig [37], DrawWing [38], and SHAPE [39]), such software packages are complicated, thus limiting its use.

Our established approach for aphid identification consists of three steps: (1) headspace collection from crushed aphids, (2) GC-MS analysis and (3) CAP analysis. This approach worked in a reproducible way and was well adapted to DNA barcoding of aphids. Notably, this methodology does work for aphids at all life stages. Moreover, it is possible to obtain further ecological insights from the aphid samples such as the degree of infestation or the geographical origin (SI, Sections S3.1–S3.8). Important next stages to develop our method as a reliable identification tool would be to either generate a larger database (dichotomous key, see Figure 10) or, better still, to develop an artificial intelligence (Al) software tool that can be used to automatically process the data. AI tools also require a large database to be trained on, so collecting more data for a database is essential. A well-established analysis of the fatty acid methyl ester profiles after lipid hydrolysis from microorganisms [28,29,30] or the analysis of MALDI protein profiles (Biotyper from Bruker) [32] for identification of microorganisms are well linked to our approach. There are several studies that make use of chemical profiles to identify aphids, e.g., based on the protein profile [23], or based on cuticular hydrocarbon analysis [76,77]. However, the key difference to our approach is that the obtained data were not subjected to CAP analysis. CAP analysis of the data allows to extract the key biomarkers for differentiation.

Clearly, standardized sampling conditions, e.g., same headspace sampling time and identical collection filters, will be required to ensure reproducibility and ease of use. In principle, miniaturized GC or GC-MS instruments [78,79] could allow aphid identification directly in the field.

With the increasing amount of data, there is a trend to establish valuable specific databases, e.g., pherobase [33], mVOC [34], GNPS [35], or MACE [36] that simplify finding relevant information, for example, about pheromones or the biological function of volatile compounds. Moreover, our approach could be extended to species identification of other organisms (arthropods). Because accurate species identification is both essential for any research and very demanding, as well as because the number of expert entomologists is unfortunately decreasing [80,81], a reliable and straightforward tool to support species identification, clearly in combination with other (morphological) methods, would be highly desirable.

## Figures and Tables

**Table 1:** Results of questionnaire performed to identify an unknown green aphids.

Questions	Answers
Aphid species?	three farmers: aphid of Taif rose two farmers: unknown five scientists: *M. rosae*
Host plants?	three farmers: Taif rose two farmers: Sultani and Taif rose five scientists: unknown
State of plant infestation?	Unknown

**Table 2:** Biomarkers of the unknown green aphids obtained by CAP analysis (Figure 12).

	Biomarkers Pattern
EB	α-Thujene (bio4) Eucalyptol (bio21) 3-Carene (bio82) Ψ-Limonene (bio84) α-Pinene (bio85) γ-Terpinene (bio86)
SEB	β-Thujene (bio10)

EB: essential biomarkers, SEB: semi-essential biomarkers.

## Data Availability

Sequencing data were deposited at the National Center for Biotechnology Information: NCBI repository (https://www.ncbi.nlm.nih.gov/nucleotide/, accessed on 5 April 2022). GenBank accession numbers.

## Supplementary Materials

The following supporting information can be downloaded at: https://www.mdpi.com/article/10.3390/insects14070589/s1, Figure S1: Maximum likelihood phylogenetic tree of the COI gene fragment of aphids. Species were collected from different sites, 36 in Taif Governorate (Kingdom of Saudi Arabia). Bootstrap values are shown based on 1000 replicates. Accession numbers of 37 isolates are shown in brackets for reference taxa (see the complete list in Table S1). Scale bar = 0.02 base 38 substitutions per nucleotide; Figure S2: GC-MS chromatograms of headspace metabolites upon cell damage of the aphid *A. punicae* (A) and its associated ant *T. magnum* (B) suggesting that 2-methyl-4-heptanone and 6-methyl-5-hepten-2-one in the profile of *A. punicae* originates from its symbiotic ant *T. magnum*; Figure S3: GC-MS chromatograms of headspace profile extracted from crushed M. rosae by CLS-charcoal (blue) and OLS-DVBP (red); Figure S4: GC chromatogram of R. porosum (A) multiple individuals, (B) one individual. The chromatograms verify the reproducibility of chemotaxonomy approach; Figure S5: GC chromatogram of *A. illinoisensis*. (A) multiple individuals, (B) one individual. The chromatograms verify the reproducibility of chemotaxonomy approach; Table S1: Aphid species, host plant, accession numbers and collection sites in Taif Governorate; Table S2: Compounds (*n* = 81) released into the headspace upon cell damage of aphids (15 samples/10 aphid species) collected from plants in Taif Governorate (Saudi Arabia); Table S3: Compounds (*n* = 11) released into the headspace upon cell damage of the unknown green aphid collected from sow-thistle (Asteraceae) in February 2022, Taif Governorate (Saudi Arabia). Metabolites were extracted by CLS and analyzed by GC-MS; Supplementary Result: S2.1: Aphid identification by phylogenetic analysis; S2.2: GC-MS analysis of headspace profiles released by aphids upon cell damage, S2.3: Preliminary multivariate analyses for chemotaxonomic differentiation of aphid species; Supplementary Discussion: S3.1: Chemotaxonomy of aphids; S3.2 Hydrocarbon profiles of aphid’s species upon cell damage; S3.3: Alcohol profiles of aphid species; S3.4: Ketone profiles of aphid species; S3.5: Ester and benzenoids in headspace profiles of aphids; S3.6: Plant-defense-related compounds in aphids; S3.7: Rapid diagnosis of aphid species by GC-MS and CAP analysis; S3.8: Comparison of headspace collection methods; S3.9: GC chromatogram of multiple individual’s vs one individual of aphid species [44,76,82,83,84,85,86,87,88,89,90,91,92,93,94,95,96].
