# Supplementary material for: Rapid Identification of Aphid Species by Headspace GC-MS and Discriminant Analysis
Source: Insects. 2023 Jun 30;14(7):589. doi: 10.3390/insects14070589 (PMC10380428; doi:10.3390/insects14070589)
Supplement: Supplementary file 1 [file insects-14-00589-s001.zip › insects-2378901-supplementary.pdf]

Table of Contents

Headlines

**Section**	page
**2. Supplementary Result**	2
2.1. Aphid identification by phylogenetic analysis	2
2.2. GC/MS analysis headspace profiles released by aphids upon cell damage	4
2.3. Preliminary multivariate analyses for chemotaxonomic differentiation of aphid species	8
**3. Supplementary Discussion**	33
3.1. Chemotaxonomy of aphids	33
3.2. Hydrocarbon profiles of aphid's species upon cell damage	33
3.3. Alcohol profiles of aphid species	34
3.4. Ketone profiles of aphid species	34
3.5. Ester and benzenoids in headspace profiles of aphids	35
3.6. Plant defense related compounds in aphids	36
3.7 Rapid diagnosis of aphid species by GC-MS and CAP analysis	37
3.8. A tool for developing an automatic aphid identification system based on chemotaxonomy	37
3.9. GC chromatogram of multiple individual's vs one individual of aphid species	39
References	40

Table of contents

**Figures**	Page
Figure S1	2
Table S1	3
Table S2	4
developed strategy to identify aphids used CLS-GC/MS-DA.	9 - 32
Figure S2	35
Table S3	37
Figure S3	38
Figure S4	39
Figure S5	39

2. Supplementary Result

2.1. Aphid identification by phylogenetic analysis.

Identified aphid species were grouped into three distinct clades: clade A contained four different species, of which *Macrosiphum rosae* L. and *Rhodobium porosum* S. were highly similar (90%) and originated from the same common ancestor, whereas *Lipaphis pseudobrassicae* D. shared 86% similarity with *M. rosae* L. and *R. porosum* S., *Aphis illinoisensis* S. originates from the same clade but had < 21% similarity. Clade B contained three species, of which *Aphis gossypii* G. and *Aphis punicae* P. were highly similar (100%), and originated from the same common ancestor, whereas *Aphis craccivora* K. shared 46% similarity with the former two. Although clades A and B evolved from the same common ancestor, sharing 83% similarity, clade C was distinct and included three species, of which *Eriosoma lanigerum* H. and *Hysteroneura setariae* T. are highly similar (88%), being derived from the same common ancestor; *Aphis nerii* was also placed in the same clade, with a similarity of 88% (Figure S1).

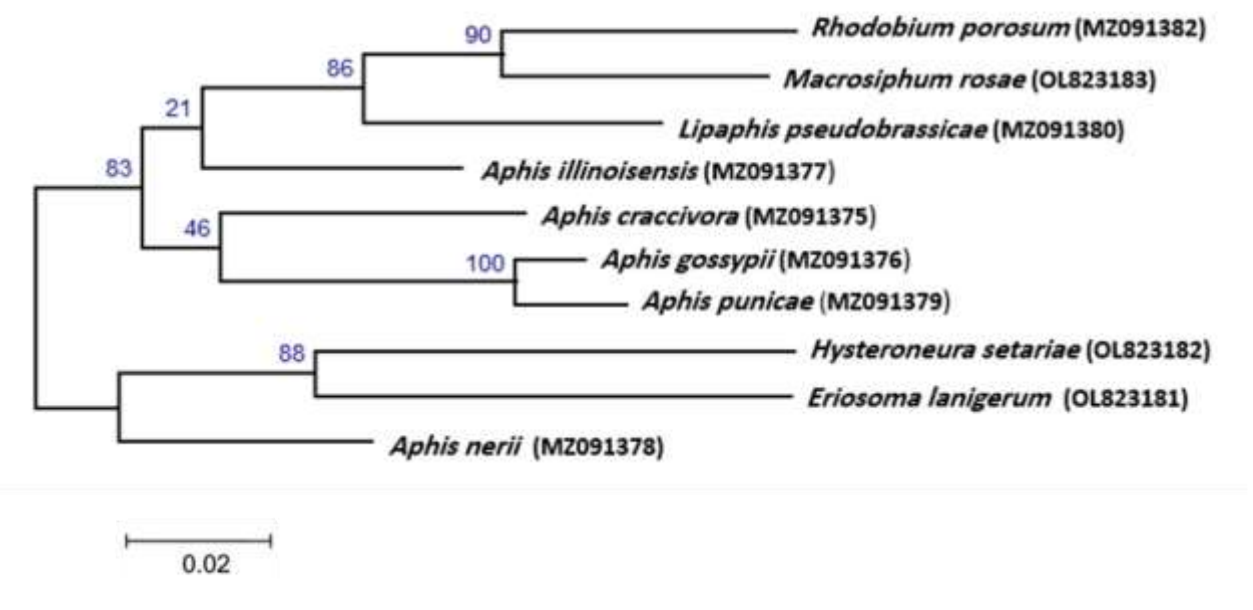

Figure S1: Maximum likelihood phylogenetic tree of the COI gene fragment of aphids. Species were collected from different sites in Taif Governorate (Kingdom of Saudi Arabia). Bootstrap values are shown based on 1000 replicates. Accession numbers of isolatesare shown in brackets for reference taxa (see the complete list in Table S1). Scale bar = 0.02 base substitutions per nucleotide

TableS1: Aphid species, host plant, accession numbers and collection sites in Taif Governorate.

Aphid samples	Aphid species	Accession No.	Host plant (plant part)	Collection site	Coordinates	Date of collection
**Pomegranate aphid**	*A. punicae* P.	MZ091379	Leaves and stems of pomegranates	Green house (Alhawia)	N21.428299, E40.473267	27-6-2021
**Cotton aphid**	*A. gossypii* G.	MZ091376	1.Leaves of zucchini	Green house (Alhawia)	N21.428299, E40.473267	21-11-2020
			2. Leaves of vinca 3. Flower buds of hibiscus plant	Alhawia	N21.428299, E40.473267	21-11-2020
			4. Leaves of lemon, 5. mint and 6. fresh Feed	Alkhalidiyah	N21.278566, E40.383781	21-5-2021
**Cowpea aphid**	*A. craccivora* K.	MZ091375	Leaves of Alfalfa	Alhawia	N21.428299, E40.473267	22-6-2021
**Rose aphid**	*R. porosum* S.	MZ091382	Leaves and stems of Taif rose	Green house (Alhawia)	N21.428299, E40.473267	22-6-2021
	M. rosae L.	OL823183		Alhada	N21.3388736, E40.3242934	11-9-2021
**Grape aphid**	*A. illinoisensis* S.	MZ091377	Leaves and stems of grape	Alsir	N21.204753, E40.591998	21-5-2021
**Oleander aphid**	*A. nerii* F.	MZ091378	Flowers of oleander	Alhawia	N21.428299, E40.473267	19-6-2021
**Woolly apple aphid**	*E. lanigerum* H.	OL823181	Stems of green apple	Alhada	N21.3388736, E40.3242934	20-8-2021
**Radish Aphid**	*L. pseudobrassicae D.*	MZ091380	Leaves of radish	Alhawia	N21.428299, E40.473267	21-11-2020
**Green foxtail aphid**	*H. setariae T.*	OL823182	Flower of green foxtail	Alsail	N21.505436, E40.493076	11-7-2021

2.2. GC-MS analysis of headspace profiles released by aphids upon cell damage.

Table S2: Compounds (n= 81) released into the headspace upon cell damage of aphids (15 samples/10 aphid species) collected from plants in Taif Governorate (Saudi Arabia). Metabolites were extracted by CLS and analyzed by GC-MS.

			**Aphids' species**	***A. punicae***	***A. illinoisensis***	***H .setariae***	***A. nerii***	***M. rosae***	***R. porosum***	***L. pseudobrassicae***	***A. craccivora***	***A. gossypii***						***E. lanigerum***	**Number of samples**
	**Biomarkers**	**RT (min)**	**Host plants-Collection site** / **Compounds**	**Pomegranate-Haw**	**Grape–AlSir**	**G. foxtail-Haw**	**Oleander-Haw**	**Taif rose-Had**	**Taif rose-Haw**	**Radish-Haw**	**Alfalfa-Haw**	**Hibiscus-Haw**	**Lemon-khal**	**Vinca-Haw**	**Mint-khal**	**Zucchini-Haw**	**Feed-Haw**	**Apple-Had**	
**monoterpenes**	Bio4	10:26	α-Thujene ?	0	+	0	0	0	0	0	0	0	0	0	0	0	0	0	**1**
	Bio5	10:45	Cyclofenchene ?	+	0	0	+	+	+	0	+	0	+	0	0	+	+	0	**8**
	Bio10	12:24	β-Thujene ?	0	0	0	0	0	0	0	0	0	+	0	0	0	0	0	**1**
	Bio19	14:77	O-Cymene ?	+	0	0	+	0	0	0	+	0	+	0	+	0	0	0	**5**
	Bio20	14:96	D-Limonene*	+	+	+	+	0	+	+	+	+	+	+	0	+	+	0	**12**
	Bio21	15:08	Eucalyptol (1,8-cineole) *	0	+	0	0	0	0	0	0	0	0	0	0	0	0	0	**1**
	Bio22	15:87	P-Cymene ?	0	0	0	+	0	0	0	0	0	0	0	0	0	0	0	**1**
	Bio33	19:45	1,3,8-P-Menthatriene ?	0	0	0	+	0	0	0	0	0	+	0	0	0	+	0	**3**
	Bio36	21:43	L-menthone ?	0	+	0	0	0	0	0	0	0	0	0	+	0	0	0	**2**
	Bio37	21:78	D-menthone ?	0	+	0	0	0	0	0	0	0	0	0	0	0	0	0	**1**
**Sesquiterpene**	Bio52	33:72	Caryophylene ?	+	+	+	+	+	+	0	+	0	0	0	0	0	0	0	**7**
	Bio53	34:47	α-Guaiene ?	0	0	0	0	+	0	0	+	0	0	0	0	0	0	0	**2**
	Bio55	35:30	Humulene ?	+	+	+	+	+	+	0	+	+	0	0	0	0	0	0	**8**
	Bio56	35:46	(*E*)-β-Farnesene*	+	+	+	+	+	+	0	+	+	+	+	+	+	+	0	**13**
	Bio59	36:75	β-Selinene ?	0	0	0	0	0	0	0	+	0	0	0	0	0	0	0	**1**
	Bio61	37:14	trans-α-Bergamotene ?	0	0	0	0	+	+	0	0	0	0	0	0	0	0	0	**2**
	Bio62	37:41	α-Bulnesene ?	0	0	0	0	0	0	0	+	0	0	0	0	0	0	0	**1**
				**6**	**7**	**4**	**8**	**6**	**6**	**1**	**9**	**3**	**6**	**2**	**3**	**3**	**4**	**0**	

**Keton**	Bio3	10:09	2-Methyl-4-heptanone ?	+	+	+	0	0	+	0	+	0	0	0	0	0	0	0	**5**
	Bio13	13:07	6-Methyl-5-hepten-2-one ?	+	+	+	+	0	+	+	+	+	0	0	0	0	0	+	**9**
	Bio57	35:65	2,6-Di-tert-butyl-p-benzoquinone ?	0	0	+	+	0	0	0	0	0	0	0	0	0	0	0	**2**
				**2**	**2**	**3**	**2**	**0**	**2**	**1**	**2**	**1**	**0**	**0**	**0**	**0**	**0**	**1**	
**Aldehyde**	Bio8	11:91	Benzaldehyde*	+	+	+	+	0	+	0	+	+	0	0	0	0	0	0	**7**
	Bio17	14:03	α-Methyl-benzeneacetaldehyde*	+	+	0	+	0	+	+	0	+	+	0	+	+	+	0	**10**
	Bio31	18:95	Nonanal*	+	+	0	+	0	+	+	+	+	+	0	0	+	+	0	**8**
				**3**	**2**	**1**	**3**	**0**	**3**	**2**	**2**	**3**	**2**	**0**	**1**	**2**	**2**	**0**	
**Esters**	Bio2	9:63	1,1-Diacetoxyethane ?	+	0	0	0	0	0	0	0	0	0	0	0	0	0	0	**1**
	Bio39	23:25	Methyl salicylate*	0	+	0	+	0	0	0	0	+	0	0	0	0	0	+	**4**
	Bio66	41:32	2,2,4-Trimethylpentane-1,3-diyl bis(2-methylpropanoate) (TXIB)??	+	+	0	0	0	0	0	0	0	0	0	0	+	+	0	**4**
	Bio77	55:60	Methyl pentacosanoate ??	+	0	0	+	0	0	0	0	0	0	0	0	0	0	0	**2**
	Bio78	55:89	Methyl hexadecanoate ??	0	+	0	0	0	0	0	0	0	0	0	0	0	0	0	**1**
	Bio81	58:84	Isopropyl palmitate ?	0	+	0	0	0	0	0	0	0	0	0	0	0	0	0	**1**
				**3**	**4**	**0**	**2**	**0**	**0**	**0**	**0**	**1**	**0**	**0**	**0**	**1**	**1**	**1**	
**Alkane**	Bio15	13:64	Decane ?	+	0	0	0	0	0	0	0	0	0	0	0	0	0	+	**2**
	Bio30	18:62	4,5-Dimethyl-nonane ??	+	+	+	+	+	+	+	+	+	+	+	+	+	+	0	**14**
	Bio40	23:69	Dodecane ??	+	+	+	+	0	+	0	+	+	0	+	0	+	+	+	**11**
	Bio43	26:88	1-Chloro-decane ?	+	+	0	0	+	+	0	+	0	0	0	0	0	0	0	**5**
	Bio44	27:26	4,6-Dimethyl-dodecane ?	+	0	0	0	0	0	0	0	0	0	0	0	0	0	+	**2**
	Bio45	28:55	Tridecane ??	+	+	+	+	0	+	0	+	0	0	0	0	0	+	0	**7**
	Bio47	29:41	Pentadecane*	0	0	0	0	0	0	0	0	0	0	0	0	0	0	+	**1**
	Bio48	29:52	4,7-Dimethyl-undecane ??	+	0	0	0	0	0	0	0	0	0	0	0	0	0	0	**1**
	Bio50	33:15	Tetradecane ??	+	+	+	+	0	+	0	+	+	+	+	+	+	+	+	**13**
	Bio60	36:97	3,8-Dimethyl-undecane ??	+	0	0	0	0	0	0	+	0	0	0	0	0	0	0	**2**
	Bio63	37:58	Hexadecane ?	+	+	+	+	0	+	0	+	0	0	+	0	+	+	0	**9**
	Bio65	38:98	Nonadecane ?	+	0	0	+	0	0	0	0	0	0	0	0	0	0	0	**2**
	Bio68	41:95	2,6,10,15-Tetramethylheptadecane ??	+	+	+	+	0	+	0	+	+	+	+	+	+	+	+	**13**
	Bio71	46:27	Heptacosane ?	+	+	+	+	0	+	0	+	+	+	+	+	+	+	0	**12**
	Bio72	48:03	Tetracosane ?	+	0	0	0	0	0	0	0	0	0	0	0	0	0	0	**1**
	Bio74	50:35	Docosane ??	0	0	0	0	0	0	0	0	0	0	0	0	0	0	+	**1**
	Bio75	50:48	Octacosane ??	+	+	+	+	0	+	0	+	+	+	+	+	+	+	0	**12**

	Bio80	58:15	Tetratetracontane ?	+	+	0	+	0	0	0	0	0	0	0	0	0	0	0	**3**
				**16**	**10**	**8**	**10**	**2**	**9**	**1**	**10**	**6**	**5**	**7**	**5**	**7**	**8**	**7**	
**Alk**	Bio67	41:50	8-Heptadecene ?	0	0	0	0	0	0	0	0	0	0	0	0	0	0	+	**1**
	Bio73	50:05	*E*-9-Eicosene ?	0	0	0	0	0	0	0	0	0	0	0	0	0	0	+	**1**
				**0**	**0**	**0**	**0**	**0**	**0**	**0**	**0**	**0**	**0**	**0**	**0**	**0**	**0**	**2**	
**Others organic**	Bio1	8:80	β-methyl-Benzeneethanamine ??	+	0	0	0	0	0	0	0	0	0	0	0	0	0	0	**1**
	Bio38	22:59	Azulene*	0	+	0	+	+	+	0	+	+	0	0	0	0	0	+	**7**
	Bio41	24:73	Benzothiazole ??	0	0	0	+	0	0	0	0	+	0	0	0	0	0	0	**2**
	Bio42	26:71	Caprolactam*	+	0	+	0	0	0	0	0	+	0	0	0	0	0	0	**3**
	Bio49	32:68	1-Tetradecanol ?	0	0	0	0	0	0	0	0	0	0	0	0	0	0	+	**1**
	Bio51	33:67	trans-Raphasatin ?	0	0	0	0	0	0	+	0	0	0	0	0	0	0	0	**1**
	Bio54	34:69	cis-Raphasatin ?	0	0	0	0	0	0	+	0	0	0	0	0	0	0	0	**1**
				**2**	**2**	**1**	**2**	**1**	**1**	**2**	**1**	**3**	**0**	**0**	**0**	**0**	**0**	**2**	
**Benzenoids**	Bio6	11:39	Bnz -6 ??	+	0	0	0	0	0	0	0	0	0	0	0	0	0	0	**1**
	Bio7	11:72	Bnz -7 ??	+	0	0	+	0	0	0	0	0	0	0	0	0	+	0	**3**
	Bio9	12:13	Mesitylene*	+	+	0	+	0	0	0	0	0	+	0	+	+	+	0	**7**
	Bio11	12:49	Bnz -11 ??	+	+	0	+	0	+	0	+	0	+	0	+	+	+	+	**11**
	Bio14	13:26	Bnz -14 ??	+	+	+	+	0	+	+	+	+	+	0	+	+	+	+	**13**
	Bio16	13:90	Bnz-16 ??	0	0	0	+	0	0	0	0	0	+	0	0	+	+	0	**4**
	Bio18	14:55	Bnz-18 ??	+	+	0	+	0	+	0	0	0	0	0	+	+	+	+	**8**
	Bio23	16:04	Bnz -23 ??	0	0	0	+	0	0	0	0	0	0	0	+	+	+	0	**4**
	Bio24	16:37	Bnz -24 ??	+	+	0	+	0	+	0	0	0	+	0	+	+	+	+	**9**
	Bio26	16:80	Bnz -26 ??	+	0	0	0	0	+	0	0	0	0	0	0	+	0	0	**3**
	Bio27	17:24	Bnz -27 ??	0	+	0	+	0	+	0	0	0	+	0	+	+	+	0	**7**
	Bio28	17:38	Bnz -28 ??	0	+	0	+	0	+	0	0	0	0	0	0	+	+	0	**5**
	Bio29	17:69	Bnz -29 ??	0	+	0	+	0	+	0	0	0	0	0	0	+	+	0	**5**
	Bio32	19:28	Bnz -32 ??	0	+	0	+	0	0	0	0	0	+	0	0	0	+	0	**4**
				**8**	**8**	**1**	**12**	**0**	**8**	**1**	**2**	**1**	**7**	**0**	**7**	**11**	**12**	**4**	
**unkno**	Bio12	12:97	UK-12	0	0	0	0	0	+	0	0	0	0	0	0	0	0	+	**2**
	Bio25	16:54	UK-25	0	0	0	0	0	+	0	0	0	0	0	0	0	0	0	**1**
	Bio34	20:72	UK-34	0	0	0	0	0	0	0	+	0	0	0	0	0	0	0	**1**

	Bio35	21:15	UK-35	0	0	0	0	0	0	0	0	0	0	0	0	+	0	0	1
	Bio46	28:86	UK-46	+	0	0	0	0	0	0	0	0	0	0	0	0	0	0	**1**
	Bio58	36:34	UK-58	+	0	0	0	+	0	0	0	0	0	0	0	0	0	0	**2**
	Bio64	38:18	UK-64	0	0	0	0	0	0	0	0	0	+	0	0	0	0	0	**1**
	Bio69	44:70	UK-69	0	0	+	0	0	0	0	0	0	0	0	0	0	0	0	**1**
	Bio70	46:16	UK-70	+	0	0	0	0	0	0	0	0	0	0	0	0	0	+	**2**
	Bio76	54:52	UK-76	+	0	0	0	0	0	0	0	0	0	0	0	0	0	0	**1**
	Bio79	57:74	UK-79	0	+	0	0	0	+	0	0	0	0	0	0	0	0	0	**2**
				**4**	**1**	**1**	**0**	**1**	**3**	**0**	**1**	**0**	**1**	**0**	**0**	**1**	**0**	**2**	
	Total biomarkers			**44**	**37**	**19**	**29**	**10**	**32**	**8**	**27**	**18**	**20**	**9**	**16**	**25**	**27**	**19**	

Compounds were identified by NIST library and RT (tentative identification). ***** Some key compounds were verified by comparison with authentic standards. 0: not detected, + detected. **Bnz**: benzene derivatives (benzenoids). **UK**: unknown compounds which were not identified by NIST library due to the reverse match below 700. Metabolites marked with a "?" had reverse match between 990-850 and marked with a "??" had reverse match between 800-700.

2.3. Preliminary multivariate analyses for chemotaxonomic differentiation of aphid species.

Closed loop stripping collected samples were analysed by gas chromatography combined to mass spectrometry followed by canonical analysis of principles coordinates (CLS-GC-MS-CAP).

The results of the molecular identification for 15 aphid samples revealed that they belonged to 10 distinct species (Figure S1).

Steps (1-21) describe the performed analysis. Steps 1-18 illustrate the five preliminary multivariate analyses (CAP A1-A5) which were conducted to separate and chemically identify 9 out of 15 different aphid species. Moreover, three further multivariate analyses (CAP B1-B3) were performed to separate the remaining six samples which all were identified as *A. gossypii* (steps 19-21) and thus the separating was based on the effect of host plants fed on by *A. gossypii*.

1- The chromatogram list after processing (smoothing window = 3 number of smooths =1 Savitzky Gollay) was copied

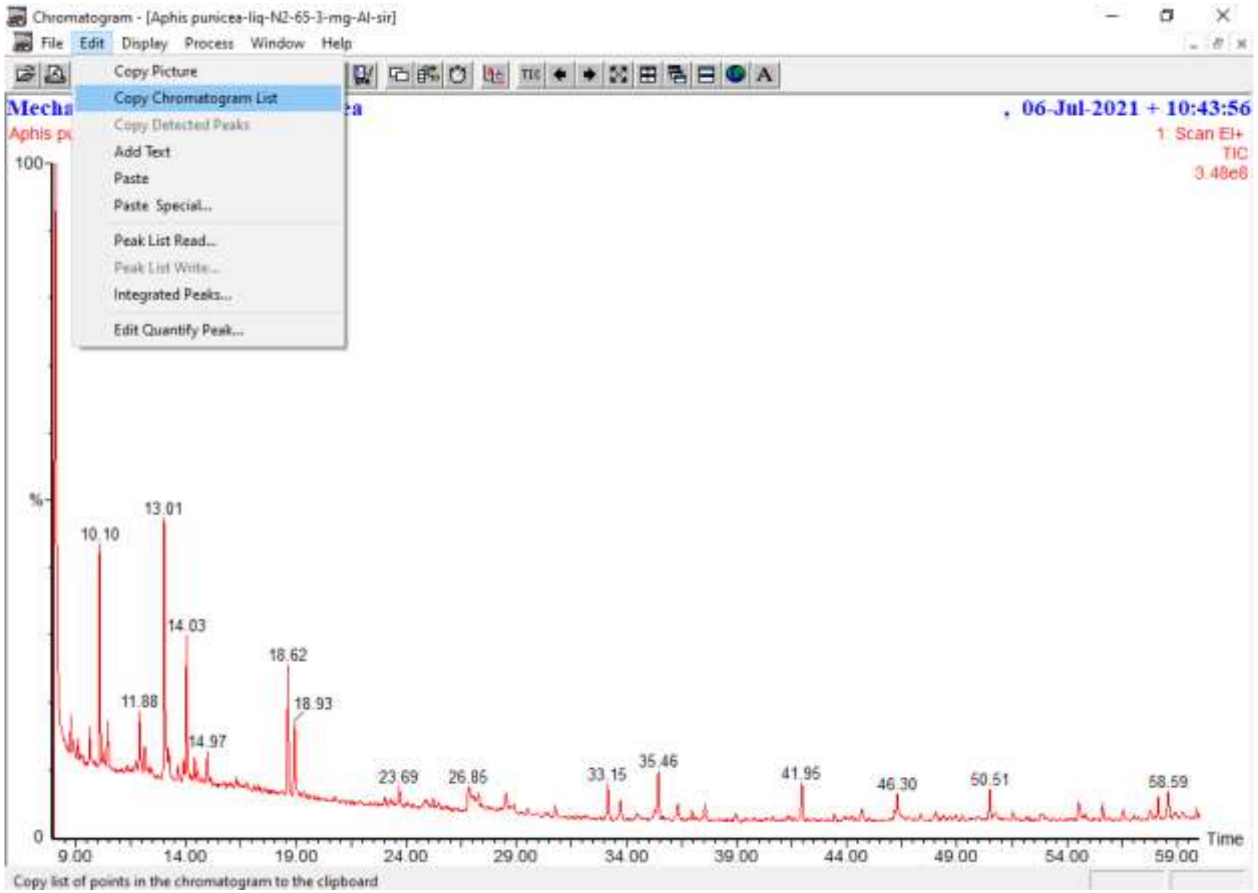

2- The chromatogram list including RT and intensity was pasted into an excel sheet. The intensities mean was calculated from 3 technical replicates of each sample collected from same host plant at same geographical site.

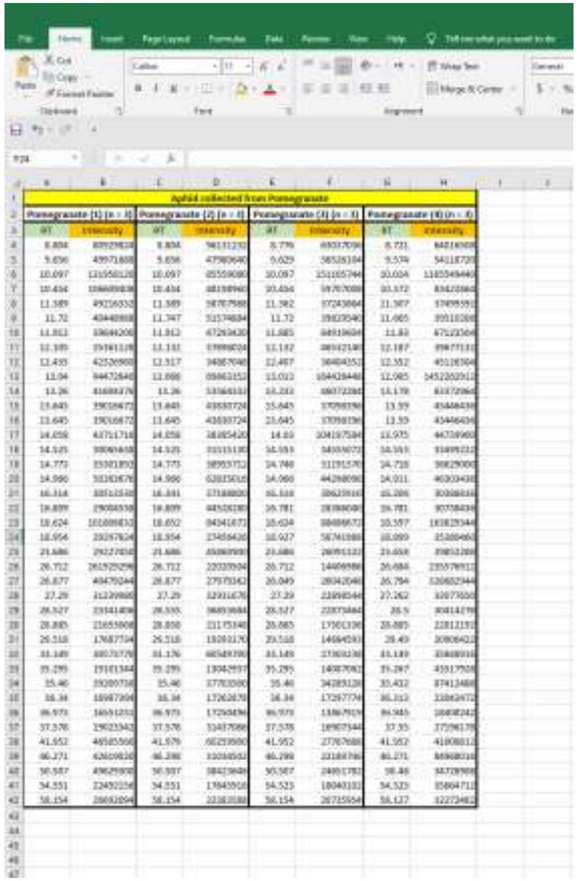

Green cells refer to the column of retention time (RT), and the **orange cells** refer to the column of intensity of each peak (n =3) detected based on the parameters considered during data collection.

3- After pasting all samples and taking the mean of their technical replicates, the mean was pasted into another excel sheet to process CAP A1

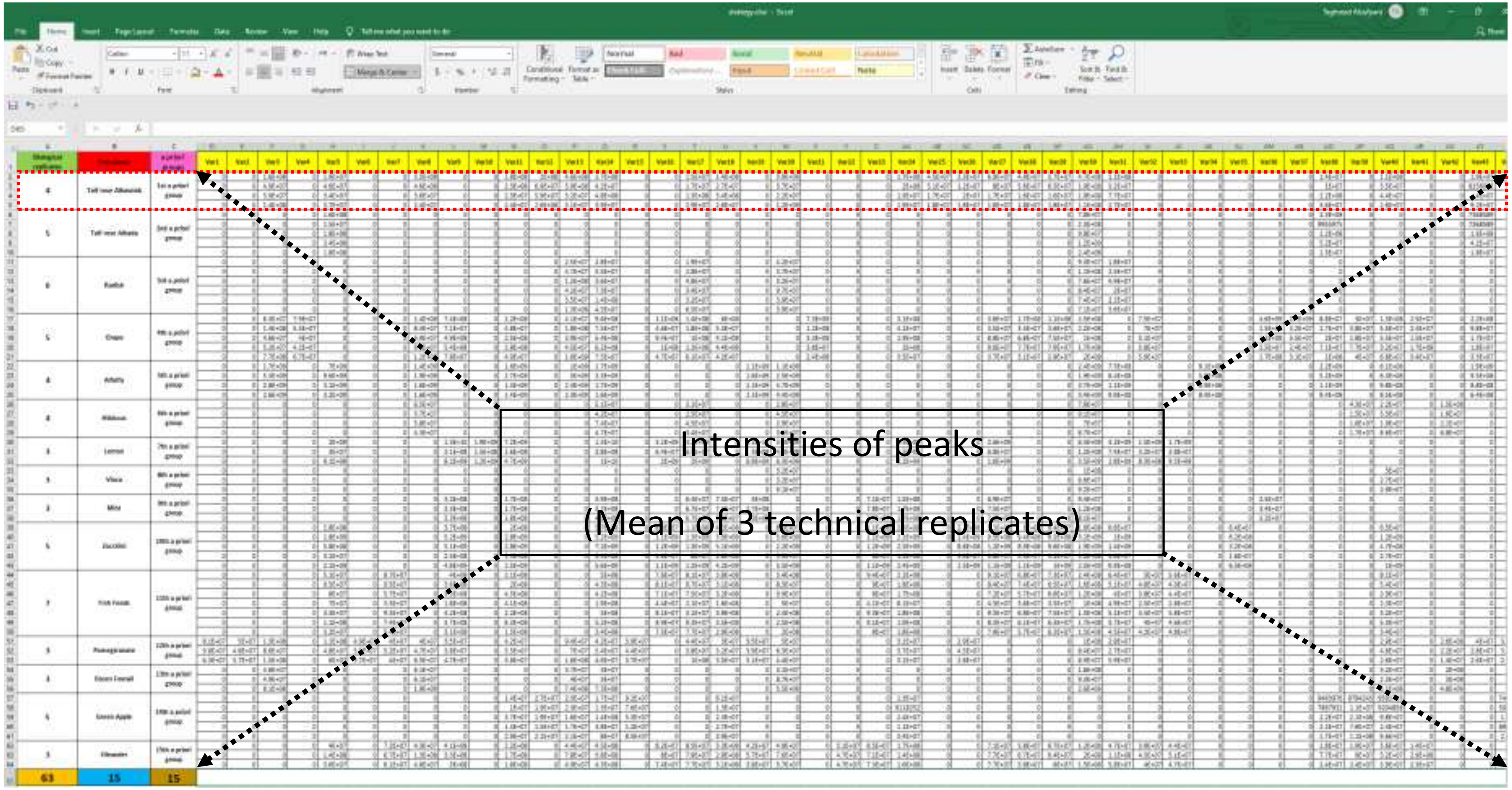

Green cells refer to the column of biological replicates (br) where br must be ≥ 3 of each sample. **Red cells** refer to the column of aphid samples based on their host plants and locations if any. **Magenta cells** refer to the a *priori* groups were suggested based on the host plants. **Orange cells** refer to the total observation equals to the sum of all biological samples (n = 63). **Blue cells** refer to the sum of samples equals to the sum of a *priori* groups (**brown cell**) which must be ≥ 3) which were considered based on host plants and locations (n = 15). The **dotted red frame** represents an example of an a *priori* group out of 15 a *priori* groups which consists of 4 biological replicates of one sample collected from same host plant. **Yellow cells** refer to the variables (peaks) detected in each sample (n = 81).

4- Only intensities were copied from the excel sheet above (step 3), pasted in a new text document and saved under a name that must be started with pk. (e.g., pk.A1.txt)

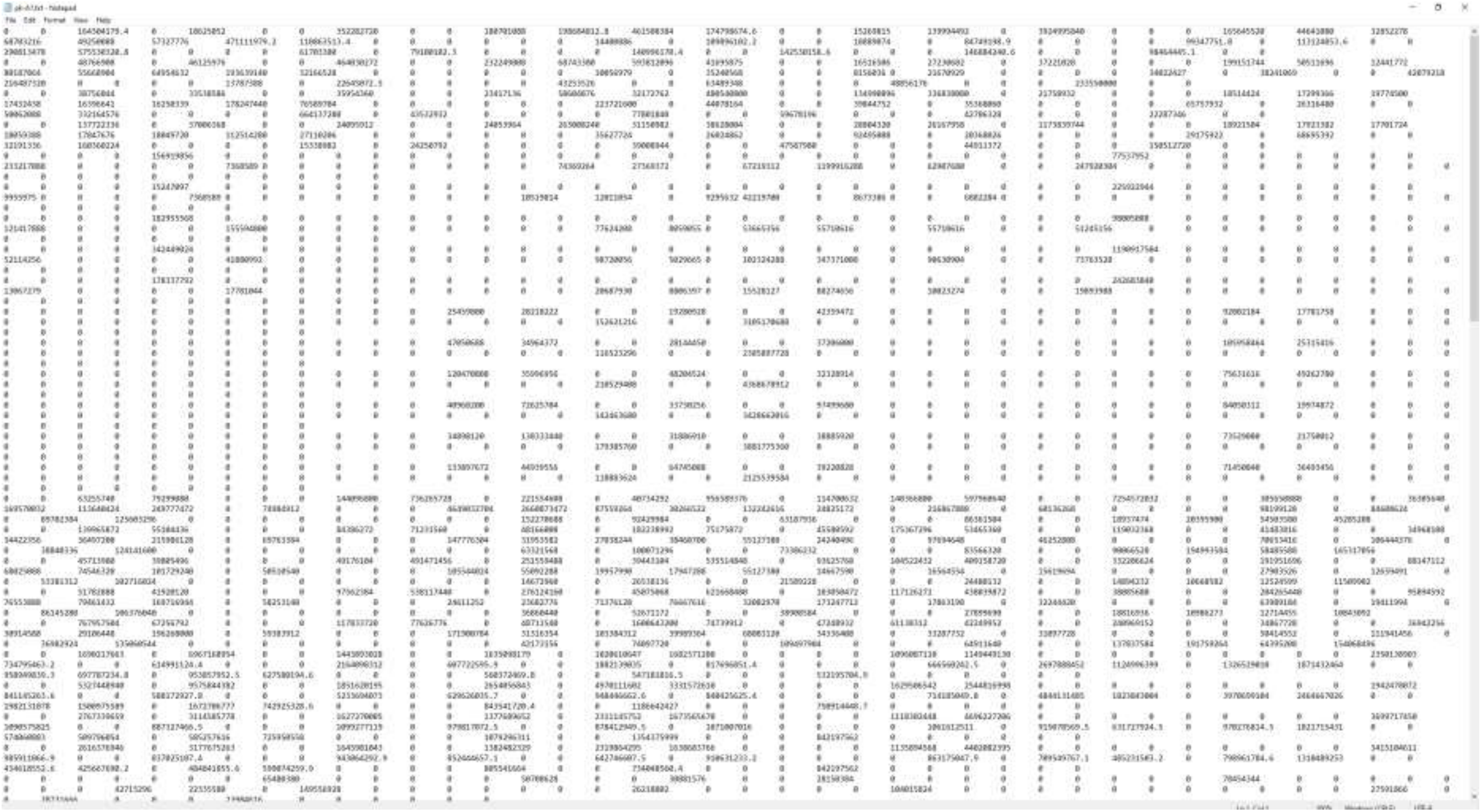

5- The free software “Canonical analysis of principle coordinates” (CAP12.exe) was used [1]The software must be located in the same folder as the last text document (pk.A1.txt) created in step 4.

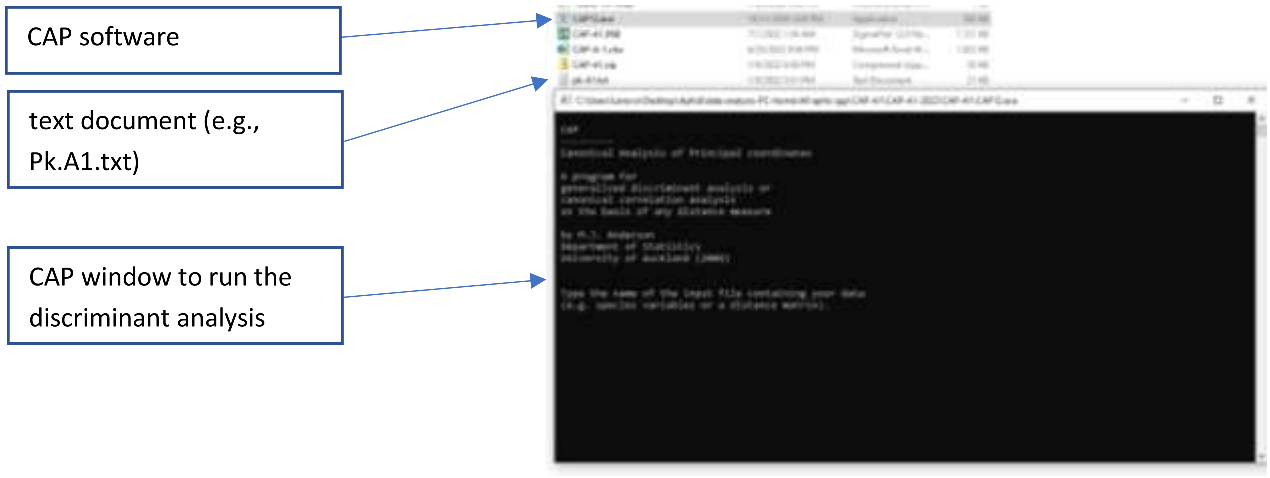

To run the CAP analysis please follow the guide manual. [1] The input file: pk.A1.txt, output file: anyname.txt (e.g.,A1.txt), nature of the data in the input file = raw data (n x p) (n= the total number of observational units and p = the number of variables), structure of the input file = rows are samples and columns are variables (see excel sheet in step 3), number of variables (columns) = 81, number of observations (rows) = number of samples = 63, no transformation, data were standardised by row (sample) sums, analysis based on Bray-Curtis dissimilarities, discriminant analysis was chosen (i.e. find axes that maximise separation among groups), numbers of groups (a *priori* groups must be ≥ 3) = numbers of host plants fed on by aphids = 15, sample size per groups = number of biological replicates in each group (i.e., 4 5 6 5 4 4 3 3 3 5 7 3 3 5 3, see the column under the green cell in step 3), let the computer program choose m (note: m is the number of PCO principal coordinate axes to be used in the canonical analysis), the principal coordinate (PCO) axes was selected and all of PCO axes were plotted by typing **999**, test by permutation was chosen, number of random permutations for the test = **9999**, an integer to be used as the seed for the random permutations = **12**, Note all bold numbers are fixed and do not affect the separation if changed.

6- The output file which was created in step 5 was opened and the data was copied and pasted into an excel sheet to analyse the result of CAP analysis based on the a *priori* groups considered in step 5.

(a)

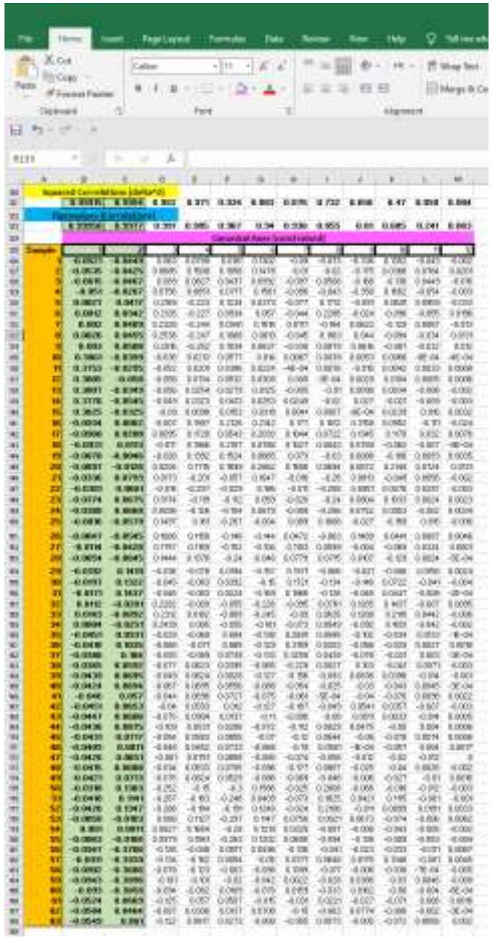

(b)

a priori groups		1	2	3	4	5	6	7	8	9	10	11	12	13	14	15	Total	%correct
Group	1	3	0	0	0	0	0	0	0	0	0	0	0	0	0	1	4	75.00%
Group	2	0	5	0	0	0	0	0	0	0	0	0	0	0	0	0	5	100.00%
Group	3	0	0	6	0	0	0	0	0	0	0	0	0	0	0	0	6	100.00%
Group	4	0	0	0	4	0	0	0	0	0	0	0	0	0	0	1	5	80.00%
Group	5	0	0	0	0	4	0	0	0	0	0	0	0	0	0	0	4	100.00%
Group	6	0	0	0	0	0	4	0	0	0	0	0	0	0	0	0	4	100.00%
Group	7	0	0	0	0	0	0	3	0	0	0	0	0	0	0	0	3	100.00%
Group	8	0	0	0	0	0	0	0	3	0	0	0	0	0	0	0	3	100.00%
Group	9	0	0	0	0	0	0	0	0	3	0	0	0	0	0	0	3	100.00%
Group	10	0	0	0	0	0	0	0	0	0	2	3	0	0	0	0	5	40.00%
Group	11	0	0	0	0	0	0	0	0	0	1	6	0	0	0	0	7	85.71%
Group	12	0	0	0	0	0	0	0	0	0	0	0	3	0	0	0	3	100.00%
Group	13	0	0	0	0	0	0	0	0	0	0	0	0	3	0	0	3	100.00%
Group	14	0	0	0	0	0	0	0	0	0	0	0	0	0	5	0	5	100.00%
Group	15	0	0	0	0	0	0	0	0	0	0	0	0	0	0	3	3	100.00%

Total correct=57/63=90.48%
Mis-calssification error= 9.52%

(a) **Yellow cells** refer to squared correlations (delta^2), **blue cells** refer to eigenvalues (correlations), **magenta cells** refer to canonical axes (m = 12 based on CAP software selection in step 5 i.e., PCO axes). **Orange column** refers to the biological samples (n = 63, see step3), **green columns** refer to the (x, y) coordinates of the first two canonical axes out of 12, which have highest delta^2 and eigenvalues. (b) Leave-one-out allocation of observations to groups (for the choice of m = 12, see step 5). **Red frame** refers to the total biological replicates in each a *priori* group. **Orange frame** refers to correct percentage to ensure if the biological replicates of each a *priori* group occupies the correct group, e.g., 1st a *priori* group has 4 biological replicates, but the correct percentage is 75% due to one of these replicates classified in 15th a *priori* group and not in 1st a *priori* group. The 10th a *priori* group has 5 biological replicates

but the %correct is 40% due to 3 out of 5 replicates classified in a different a *priori* group which is 11th a *priori* group. **Green frame** refers to total correct and miss classification error of a *priori* groups that was considered in step 3.

7- 7- The coordinates (x, y) of the first two canonic axes of the 63 samples (the green columns in step 6) have been pasted into the SigmaPlot worksheet. From the creation of the graph, a simple scatterplot was chosen, then the first group a priori (see step 3) was selected to be plotted, the other 14 groups were plotted sequentially one by one by choosing add new plot in the graph page.

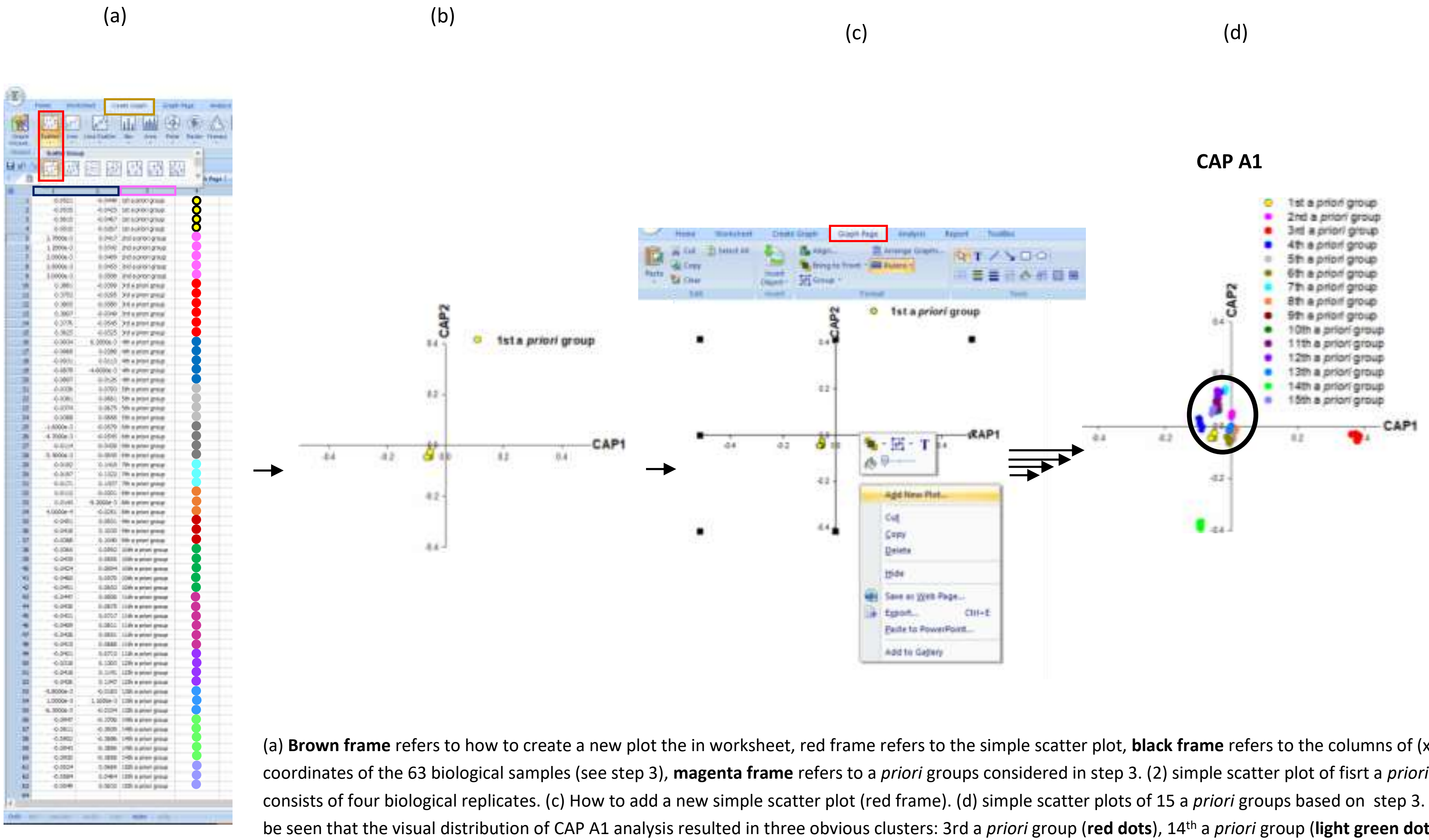

(a) **Brown frame** refers to how to create a new plot the in worksheet, red frame refers to the simple scatter plot, **black frame** refers to the columns of (x, y) coordinates of the 63 biological samples (see step 3), **magenta frame** refers to a *priori* groups considered in step 3. (2) simple scatter plot of fisrt a *priori* group consists of four biological replicates. (c) How to add a new simple scatter plot (red frame). (d) simple scatter plots of 15 a *priori* groups based on step 3. It can be seen that the visual distribution of CAP A1 analysis resulted in three obvious clusters: 3rd a *priori* group (**red dots**), 14th a *priori* group (**light green dots**), and the other remaining a *priori* groups surounded by a black circle. Based on the visual inspection of the distribution (d), we decided that the mis-classification error which was = 9.52 (step 6-(b)) was not significant to represent the defrences between the a *priori* groups. Thus, further CAP A2 with only 3 a *priori* groups was applied.

8- In an excel sheet similar to that in step 3 but with changing the a *priori* groups from 15 to only 3 a *priori* groups so the order of groups had to be changed in order to combine the groups of each individual a *priori* group together.

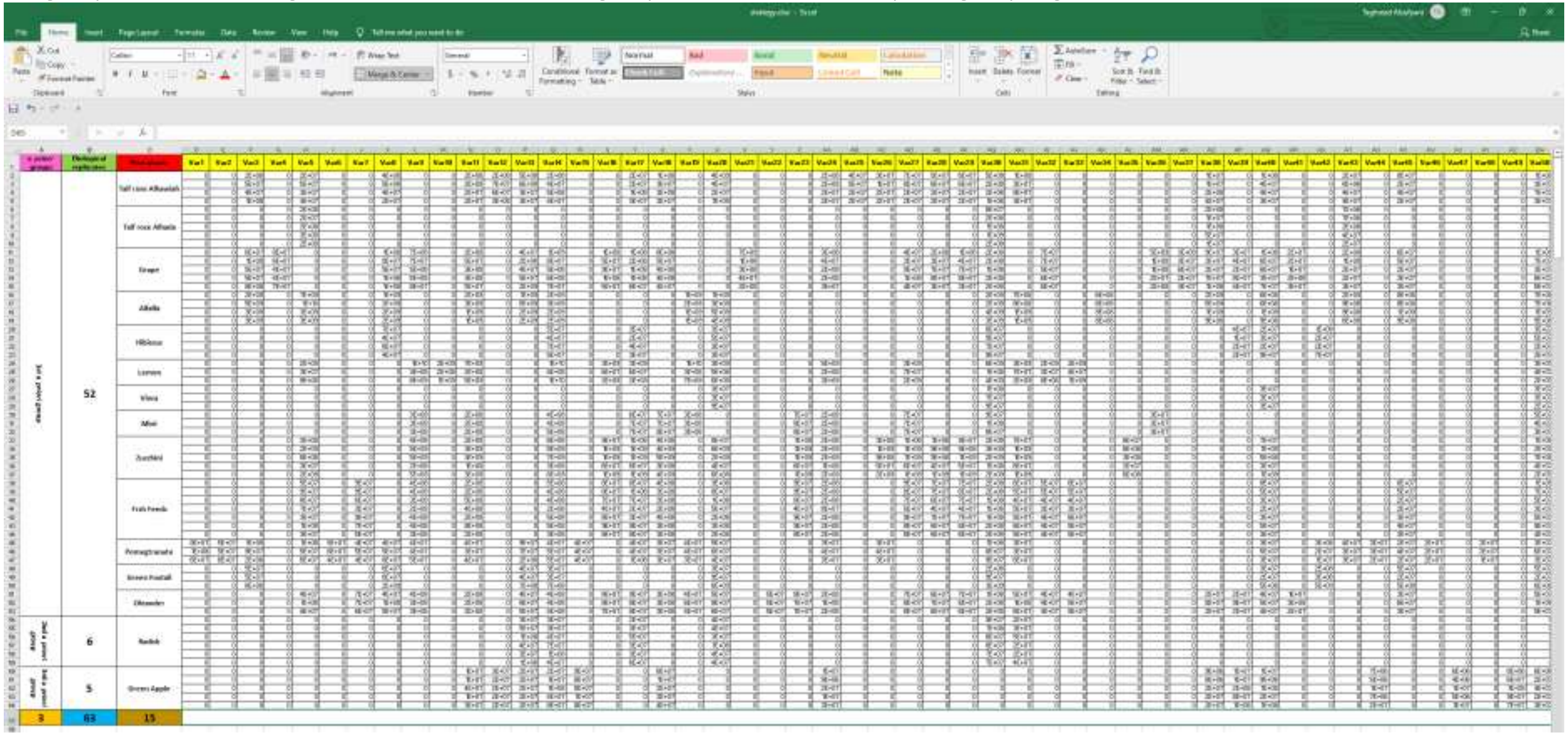

Magenta cells refers to the a *priori* groups considered based on our observation in step 7. **Green cells** refer to the column of biological replicates of each a *priori* group (i.e., 1st a *priori* group has 52 biological replicates, 2nd a *priori* group has 6 biological replicates, 3rd a *priori* group has 5 biological replicates). **Red cells** refer to the column of aphid samples based on their host

plants and locations if any. **Yellow cells** refer to the variables (peaks) detected in each sample (n = 81). **Orange cell** refers to the total a *priori* groups (n = 3). **Blue cell** refers to the total observations that equal to the sum of all biological samples (n = 63). **Brown cell** refers to the sum of samples considered based on host plants and locations (n = 15).

9- We followed the same steps from 3 to 6 to carry out CAP A2 with a special consideration when we processed CAP analysis in step 5. The numbers of groups i.e., a *priori* groups = 3, sample size per groups = number of biological replicates in each group (i.e., 52, 6, 5, see the column under the green cell), and started running the CAP analysis to obtain the output file which was copied and pasted in excel sheet to start SigmaPlot to visualize the data by a simple scatter plot.

(a)

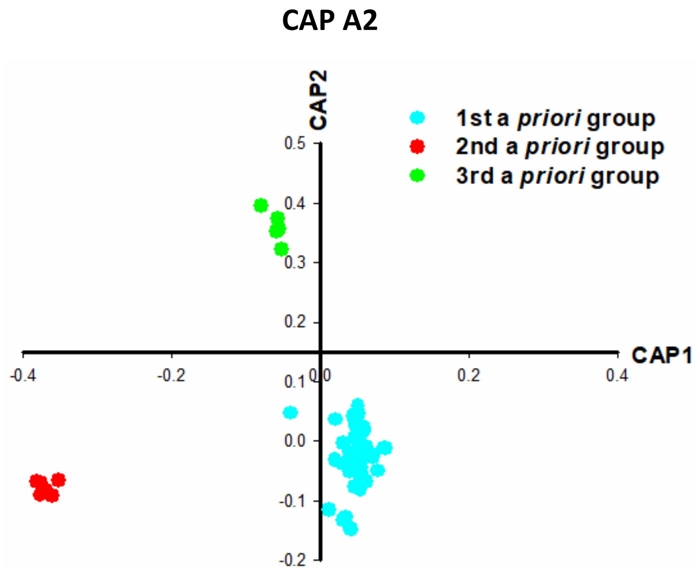

(b)

a priori groups		1	2	3	Total	%correc
Group	1	52	0	0	52	100.00%
Group	2	0	6	0	6	100.00%
Group	3	0	0	5	5	100.00%
Total correct = 63/ 63		100.00%				
Mis-classification error		0.00%				

(a) Simple scatter plot resulted in a clear visual distribution of the 3 a *priori* groups of the 63 biological samples (see the column under the green cell in step 8). (b) mis-classification error is 0.0% (**red frame**) and each a *priori* group has its all-biological replicates.The percentage of correct of each suggested a *priori* group is 100% (**green frame**).

10- As the visual distribution of the 3 a *priori* groups in step 9 was clear with mis-classification error (0.0%), the vector plot was curried out to visualize the variables (*n* = 81). The number of peaks were detected in all 63 samples which were plotted in step 9. From the output file created in step 10, the two first axes which represent the correlations of canonical axes plotted in step 10 (1) with original variables (*n* = 81; i.e., peaks detected in all 63 samples) were copied and pasted in the same worksheet of SigmaPlot used in step 10.

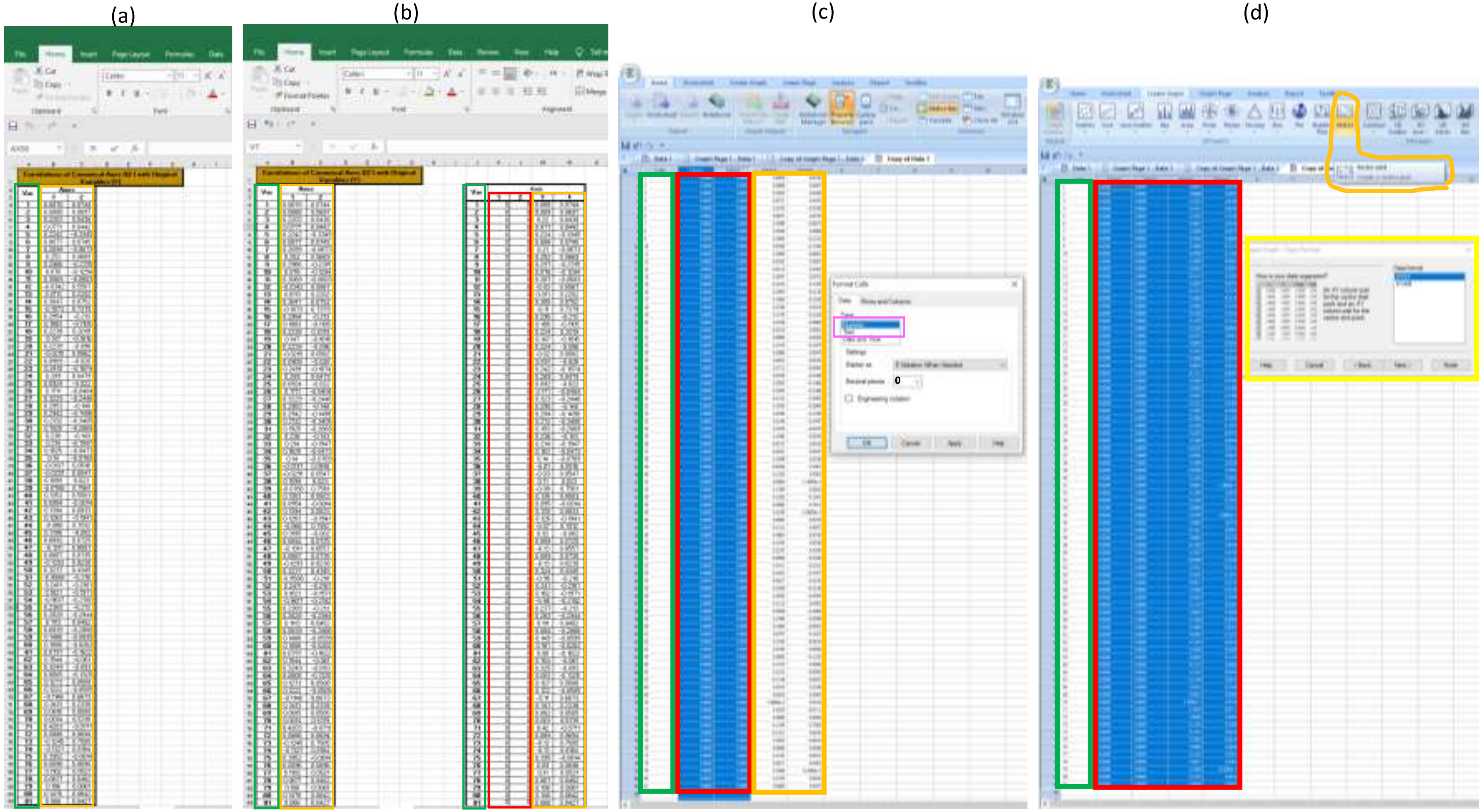

(e)

CAP A2

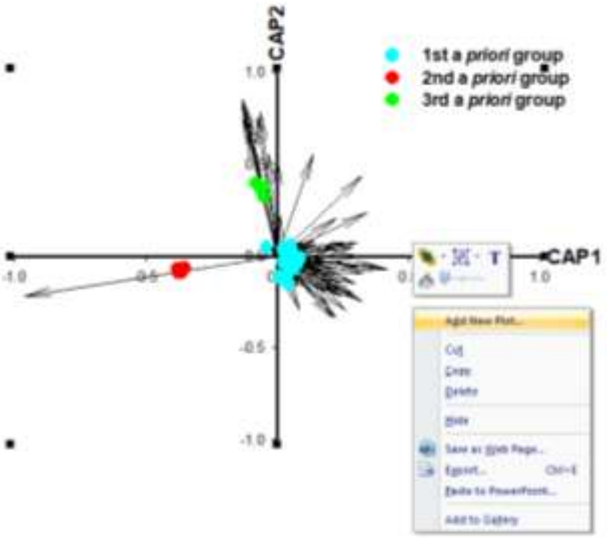

(a) The original data represents the variables (*n* = 81, **green frame**) and first two axes (**orange frame**) which correlate to the canonical axes used in step 10 with the 81 variables (**green frame**). (b) For further process, the two axes considered as (x_2, y_2) coordinates (**orange frame**) and (x_1, y_1) coordinates (**red frame**) were added as (0, 0) for the 81 variables (**green frame**). The variables (**green frame**) and the coordinates (x_1, y_1) (**red frame**)/ (x_2, y_2) (**orange frame**) in (b) were copied and pasted in SigmaPlot worksheet (c). After that, variables (**green frame**) were converted to 0 decimals numeric data and text by format cells (**magenta frame**). In create graph (d), vector plot (**orange frame**) was selected with data format (xyxy) (**yellow frame**) and all coordinates in **red frame** were selected once to get vectors (arrows) separating the a *priori* groups (e). To detect which variable represents each vector in (e), add new plot was selected to draw simple scatter plot and only the first two axes (**orange frame** in (c)) were selected.

11- After the simple scatter plot, figure (e) looks like as figure (f). Now, we can realize which variable participates in separating each isolated a *priori* group. The longer arrow the more significant vector in characterizing the isolated a *priori* group. In CAP A2 (f), three a *priori* groups were separated clearly: 1st a *priori* group (**light blue dots**) consists of 13 samples, 2nd a *priori* group (**red dots**) consists of 1 sample, and 3rd a *priori* group (**green dots**) consists of 1 sample. We interested in isolated a *priori* group only when it consists of one sample. We now knew which vector(s) (variable(s)) out of 81 contribute(s) in separating and characterizing 2nd and 3rd a *priori* groups. The sample of 2nd and 3rd a *priori* groups underwent DNA analysis to ensure that they are different aphid species.

Molecular identification revealed that 2nd a *priori* group is *Lipaphis pseudobrassicae*, and 3rd a *priori* group is *Eriosoma lanigerum*. The 1st a *priori* group consisting of 13 samples is characterized by (*E*)-β-farnesene (vector n# 56). All the 13 aphid species in the 1st a priori group released (*E*)-β-farnesene, but not theaphid species of 2nd and 3rd a *priori* groups.

(f)

CAP A2

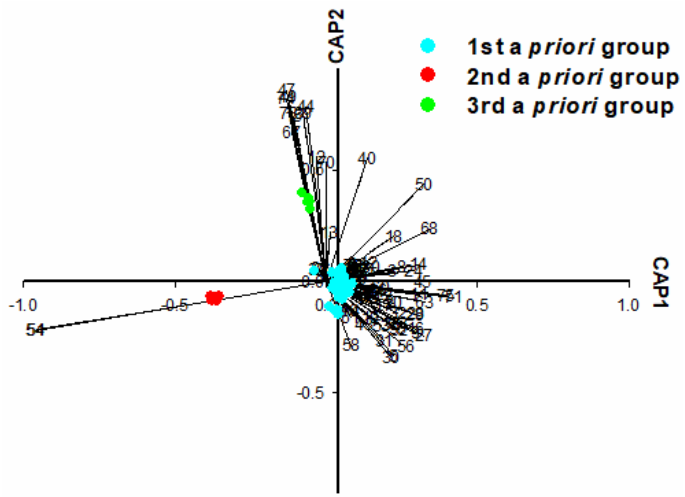

In CAP A2, 2nd a *priori* group: *L. pseudobrassicae* (**red dot**) is characterized by vectors (variables) #51, and #54. 3rd a *priori group: E lanigerum* (**light green dot**) is characterized by #13, and #15, #38, #39, #40, #49, #50, #67, #68, #73, and #74.

12- The 1st a *priori* group was subjected to further CAP analysis because this a *priori* group consisted of 13 samples, and it must be distributed to individual a *priori* groups to chemically identify the aphid species which are represented by the 1st a *priori* group. To do so, the same excel sheet in step 3 was used but the 3rd and 14th a *priori* groups were removed (which correspond to the 2nd and 3rd a *priori* groups, respectively in step 8), as well as all variables belonging to these a *priori* groups were removed. Thus, the variables changed from 81 to 74. The a *priori* groups = 13 and the observations (samples) = 13.

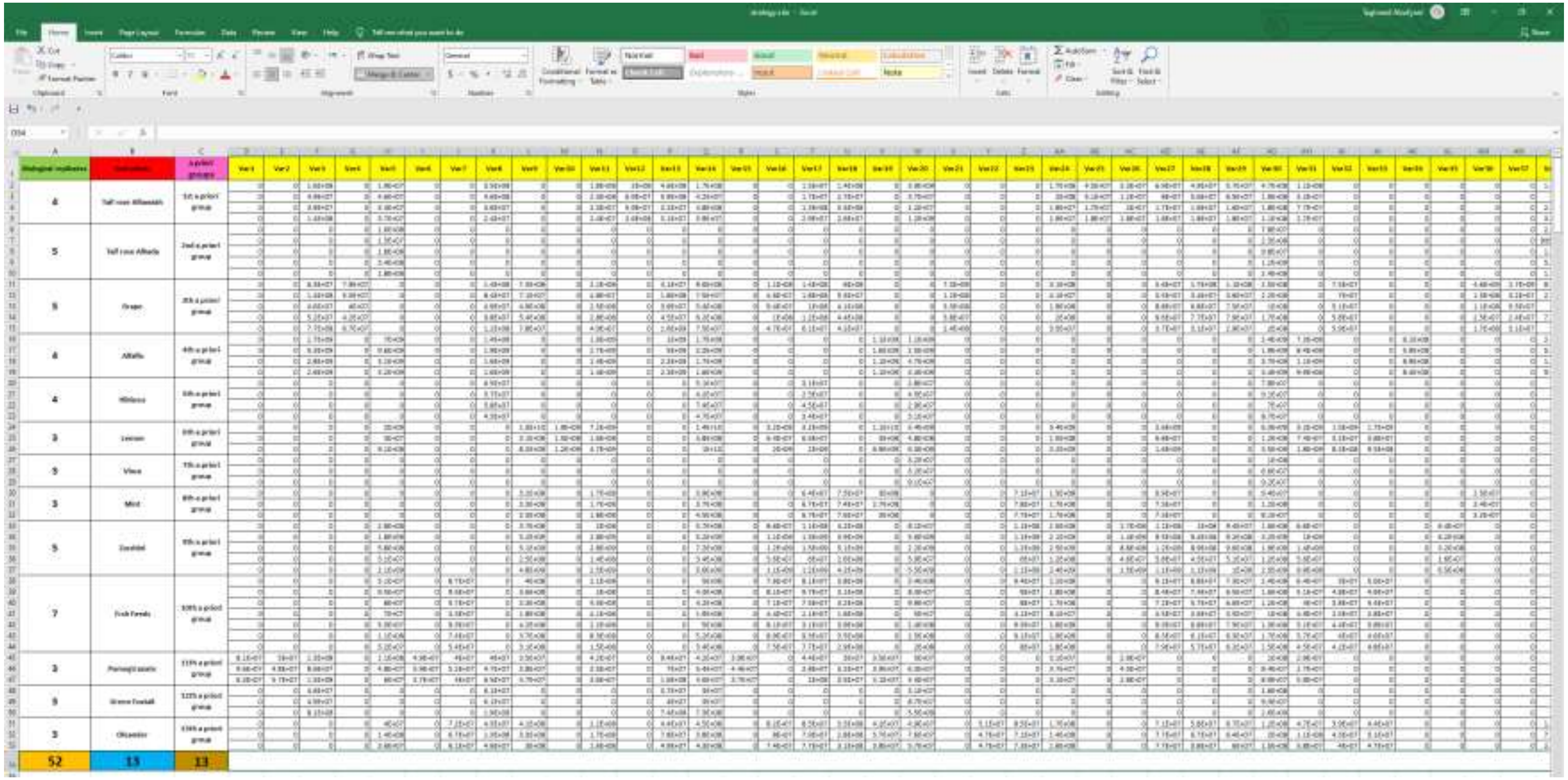

13- Following the same steps from 3 to 6 considering the information mentioned in step 13. The output file was visualized using SigmaPlot

(a)

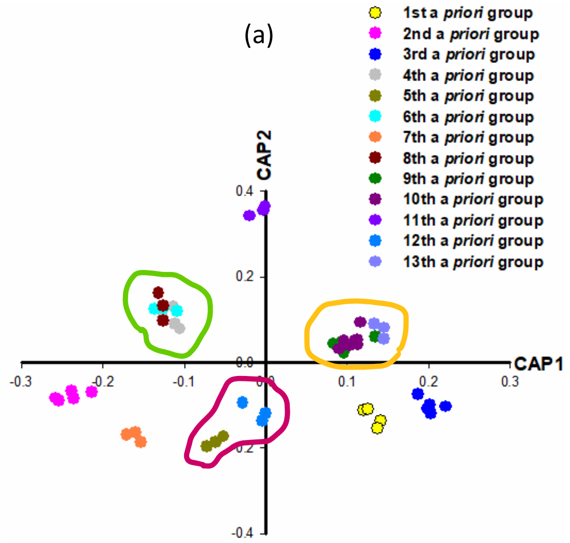

(b)

a priori groups		1	2	3	4	5	6	7	8	9	10	11	12	13	Total	%correct
Group	1	2	0	1	0	0	0	0	0	0	0	0	0	1	4	50.00%
Group	2	1	3	0	1	0	0	0	0	0	0	0	0	0	5	60.00%
Group	3	0	0	4	0	0	0	0	0	0	0	0	0	1	5	80.00%
Group	4	0	0	0	4	0	0	0	0	0	0	0	0	0	4	100.00%
Group	5	0	0	0	0	2	0	1	0	0	0	0	1	0	4	50.00%
Group	6	0	0	0	0	0	3	0	0	0	0	0	0	0	3	100.00%
Group	7	0	0	0	0	0	0	3	0	0	0	0	0	0	3	100.00%
Group	8	0	0	0	0	0	0	0	3	0	0	0	0	0	3	100.00%
Group	9	0	0	0	0	0	0	0	0	4	1	0	0	0	5	80.00%
Group	10	0	0	0	0	0	0	0	0	0	7	0	0	0	7	100.00%
Group	11	0	0	0	0	0	0	0	0	0	0	3	0	0	3	100.00%
Group	12	0	0	0	0	0	0	0	0	0	0	0	3	0	3	100.00%
Group	13	0	0	0	0	0	0	0	0	0	0	0	0	3	3	100.00%

Total correct = 44/ 52 = 84.615%
Mis-classification error = 15.385%

(a) Simple scatter plot of 13 a priori groups. The visual distribution is not sufficient. (b) The mis-classification error is 15.4% (**red frame**) and the biological replicates of some a *priori* groups are not placed in the correct group (**red arrows**). Overall, the CAP analysis was rejected due to the value of misclassification error and thus the classification of the a *priori* groups were changed. It can be clearly observed in (1) that 4th, 6th, and 8th a *priori* groups (**green frame**) formed one cluster, 9th, 10th, and 13th a *priori* groups formed another cluster (**orange frame**), and 5th and 12th a *priori* groups formed a cluster (**dark magenta frame**)

14- From the above observation the 13 a *priori* groups in step 13 changed to 8 in this step as follows: all first 5 a *priori* groups stayed individual represent only one sample/each, the 6th a *priori* group consisted of 3 samples (the pervious 4th, 6th, and 8th a *priori* groups in steps 12, 13), 7th a *priori* group consists 3 samples (the pervious 9th, 10th, and 13th a *priori* groups in steps 12, 13), and 8th a *priori* group consisted of 2 samples (the pervious 5th, and 12th a *priori* groups in steps 12, 13). See the excel sheet below (a). The data was analysed by CAP (n# of variables = 74, n# of observation = 52), and visualized by SigmaPlot. A very random distribution was obtained (b).

(a)

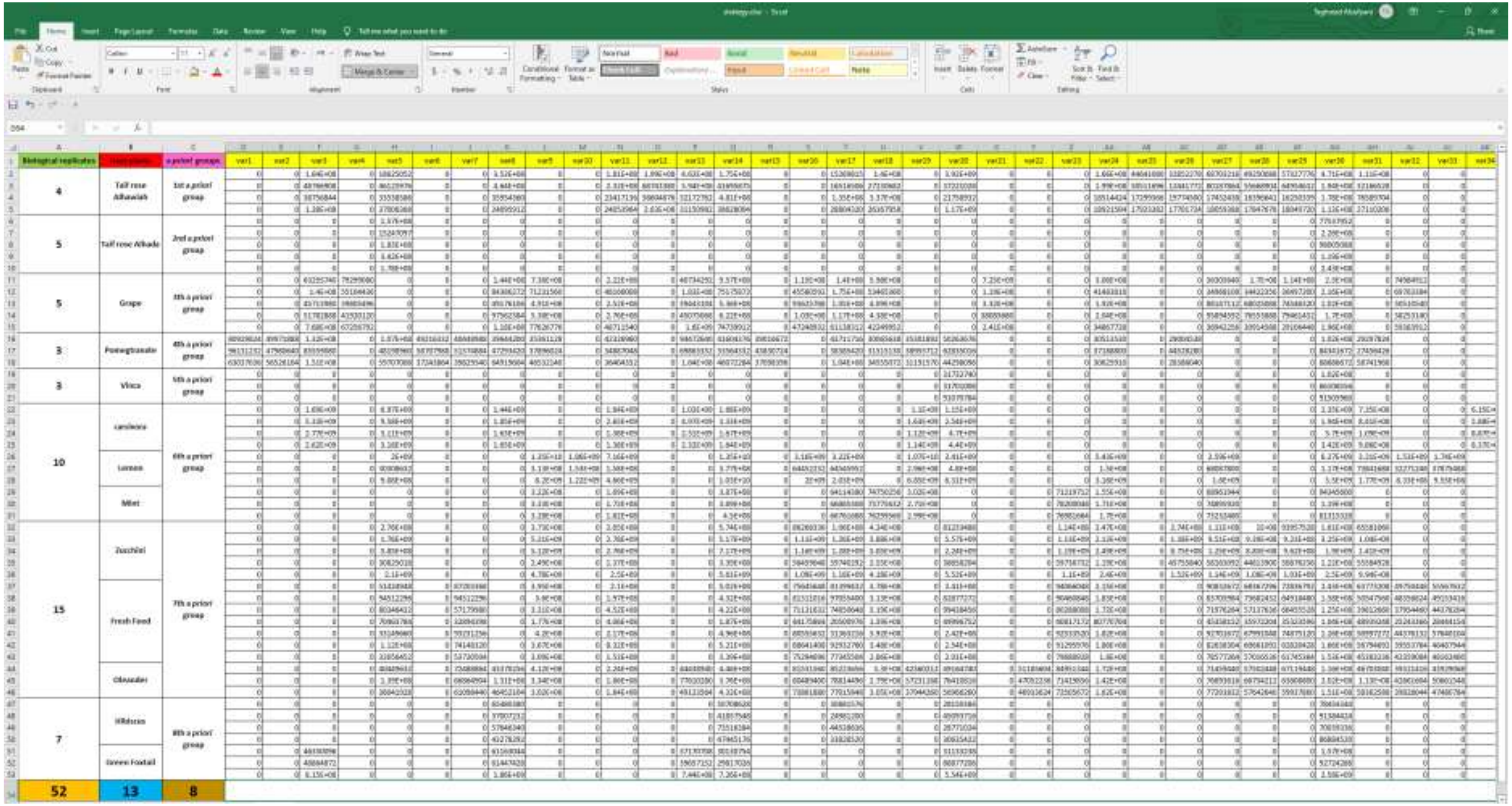
a priori groups
var1
4
Tall rose
5
Grape
3
Pomegranate
Vinca
10
Lemon
15
Zucchini
Fresh Food
7
Green Foxtail
52
13
8

(b)

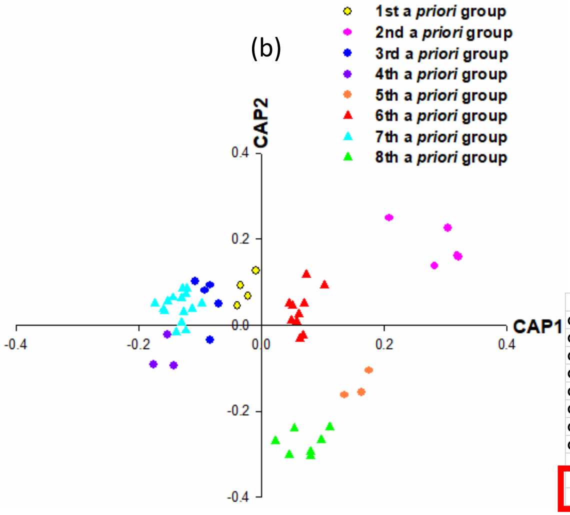

(c)

a priori groups		1	2	3	4	5	6	7	8	Total	%correct
Group	1	3	0	0	0	0	0	0	1	4	75.00%
Group	2	0	5	0	0	0	0	0	0	5	100.00%
Group	3	0	0	4	0	0	0	0	1	5	80.00%
Group	4	0	0	0	3	0	0	0	0	3	100.00%
Group	5	0	0	0	0	10	0	0	0	10	100.00%
Group	6	0	0	0	0	0	7	0	0	7	100.00%
Group	7	0	0	0	0	0	0	3	0	3	100.00%
Group	8	0	0	0	0	0	0	0	15	15	100.00%

Total correct = 50/ 52 = 96.154%
Mis-classification error = 3.846%

(d)

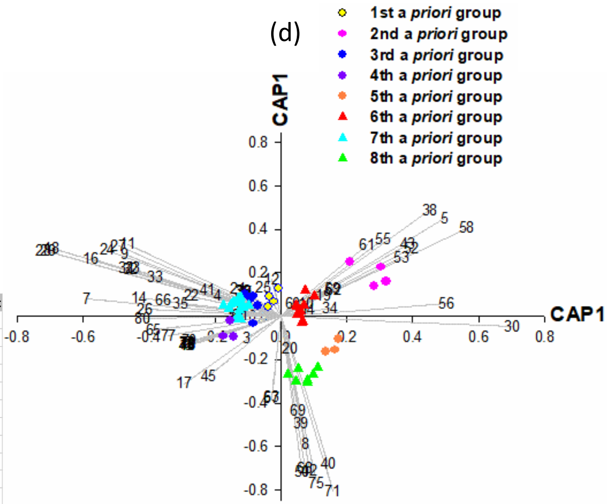

(b) Simple scatter plot of 8 a priori groups, no clear visual distribution was obtained, although the mis-classification error in (c) = 3.85% (**red frame**). Here, an important decision (the result will be accepted or rejected) was taken after we plotted the vectors (d). No vector(s) was detected as a characteristic variable to any individual a *priori* group i.e., 1st, 2nd, 3rd, 4th, and 5th because the nature of distribution. Even worse, the last individual a *priori* group (**orange dots**) was not characterized by any vector although it was fully separated. That is, the result was rejected. Different classifications of a *priori* groups were applied with special consideration that the number of variables is fixed to 74 (see step 12).

15- After different attempts of classification, the best visual distribution with significant mis-classification error and percentage corrects of individual a *priori* groups led to CAP A3. Four out of five a *priori* groups were individuals consisting only of 1 sample, except the 5th a *priori* group consisting of 9 samples of aphid species (the pervious 1st, 3rd, 5th, 6th, 7th, 8th, 9th, 10th, and 13th a *priori* groups in steps 12, 13). Phylogenetic analysis of the four individuals a *priori* groups revealed that the 1st a *priori* group is *Macrosiphum rosae*, 2nd a *priori* group is *Aphis craccivora*, 3rd a *priori* group is *Aphis punicae*, 4th a *priori* group is *Hysteroneura setariae*. The 5th a *priori* group was not molecularly identified because it consisted of 9 samples.

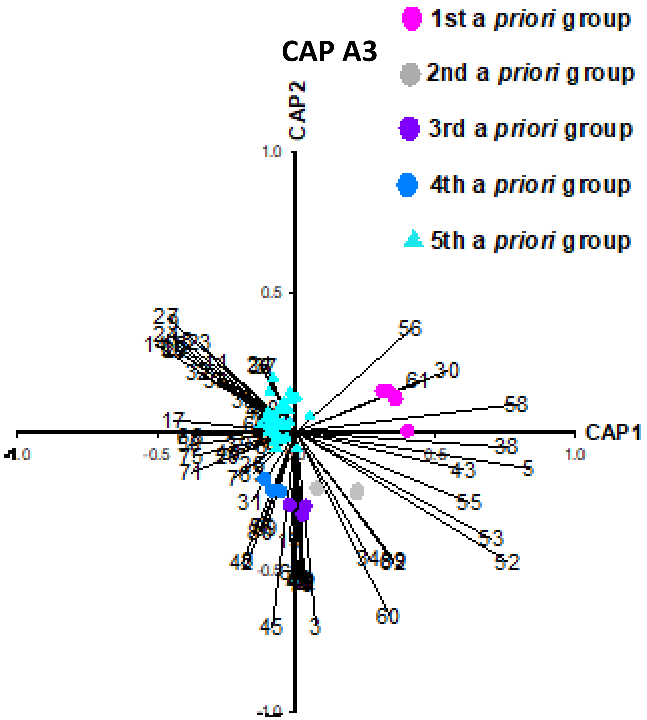

In CAP A3, four *a priori* groups were isolated and characterized by specific vectors (variables). 1st a *priori* group: *M. rosae* (**magenta dots**) is characterized by vectors #58, #38, #30, and #61. 2nd a *priori* group: *A. craccivora* (**grey dots**) is characterized by #60, #59, #62, and #34. 3rd a *priori* group: *A. punicae* (**purple dots**) is characterized by #45, #3, #1, #2, #6, #13, #15, #44, #45, #46, #48, #65, #70, #72, #76, and #77. 4th a *priori* group: *H. setariae* (**dark blue dots**) is characterized by vectors #8, #42, #57, #69, #80, and #31. The 5th a *priori* group (**light blue triangles**) was subjected to further CAP analyses because it consists of 9 samples.

16- The 5th a *priori* group in step 15 was organized to obtain a clear separation of the 9 samples. A suggestion wording was performed to apply CAP analysis on 9 a *priori* groups (a) resulted in mis-classification error = 13.5% (b).

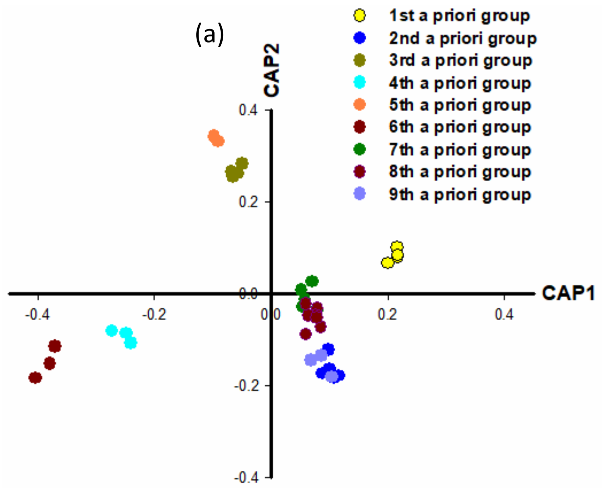

(b)

a priori groups		1	2	3	4	5	6	7	8	9	Total	%correct
Group	1	2	1	0	0	0	0	0	1	0	4	50.00%
Group	2	0	4	0	0	0	0	0	0	1	5	80.00%
Group	3	0	0	4	0	0	0	0	0	0	4	100.00%
Group	4	0	0	0	3	0	0	0	0	0	3	100.00%
Group	5	0	0	0	0	3	0	0	0	0	3	100.00%
Group	6	0	0	0	0	0	3	0	0	0	3	100.00%
Group	7	0	0	0	0	0	0	5	0	0	5	100.00%
Group	8	0	0	0	0	0	0	1	6	0	7	85.71%
Group	9	0	0	0	0	0	0	0	1	2	3	66.67%

Total correct = 32/ 37 = 86.486%
Mis-classification error = 13.514%

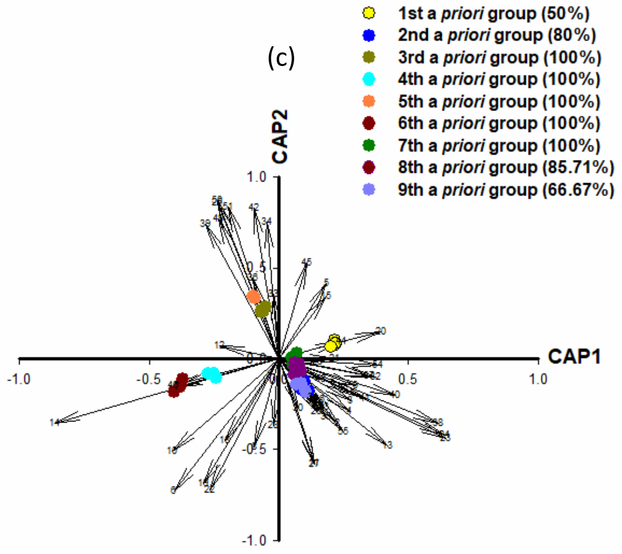

(a) A simple scatter diagram of 9 a priori groups indicates that 100% isolated a priori groups are the 3rd, 4th, 5th, 6th and 7th. (b) The misclassification was high (red box) in comparison with all accepted CAP analyses. (c) For evidence, vectors were plotted, and it can be obviously seen that vectors are overlapping and as a result there is no a *priori* group that possesses a vector that distinguishes it. Thus, this CAP analysis was rejected, and further CAP analyses were carried out.

17- After different attempts to have 100% isolated as well as individual a *priori* group(s) with characteristic vectors for the isolated groups. The best distribution was obtained by some modification in the classification of the a *priori* groups in step 16. The 1st and 2nd a *priori* groups were combined as well as the 3rd – 8th a *priori* groups. The only individual a *priori* group is the 9th a *priori* group. Thus, in CAP A4 there are 3 a *priori* groups as follows: 1st a *priori* group (the pervious 1st, and 2nd a *priori* groups in step 16a, c), 2nd a *priori* group (the pervious 3rd, 4th, 5th, 6th, 7th, and 8th a *priori* groups in step 16a, c), and 3rd a *priori* group represents 1 sample (the pervious 9th a *priori* groups in step 16a, c). Molecular identification revealed that this sample (**light purple dot**) is *Aphis nerii*.

(a)

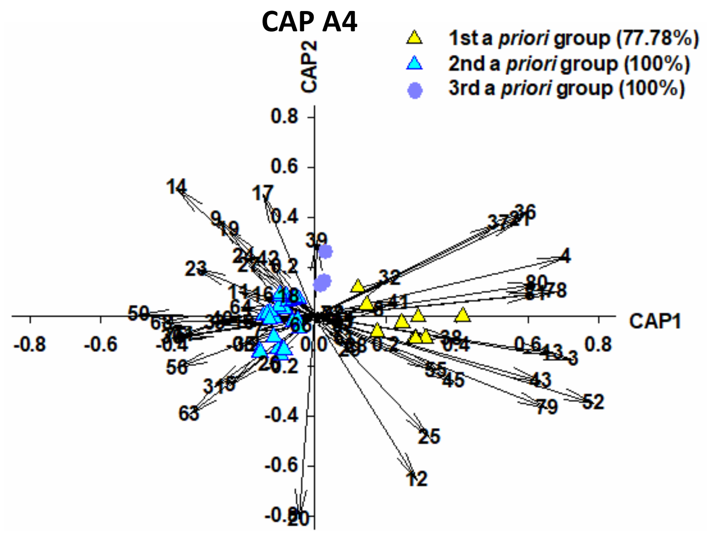

(b)

a *priori* groups		1	2	3	Total	%correct
Group	1	7	2	0	9	77.78%
Group	2	0	0	25	25	100.00%
Group	3	0	3	0	3	100.00%
Total correct = 35/37 = 94.60%						
Mis-classification error = 5.41%						

(a) Simple scatter and vector plots of CAP A4 resulted in acceptable visual distribution and only one individual isolated a *priori* group i.e., 3rd a *priori* group (**light purple dots**): *A. nerii* which was distinguished by vector #39. 1st and 2nd a *priori* groups were subjected to CAP analysis to be separated. (b) Mis-classification error equals to 5.41%.

18- The 1st group a priori of step 17 consists of 2 samples and was therefore submitted for analysis CAP A5. In that case, a particular consideration should be taken into account.. As it was mentioned in steps 3-5, the suggested a *priori* groups in any CAP analysis must be equal to 3 or more. The 1st a *priori* group in step 17 consists of 2 samples only so the a *priori* groups will be 2 which is not acceptable to carry out CAP analysis. Thus, an outgroup was added to increase the number of a *priori* groups from 2 to 3. The

suggested a *priori* groups are 1st a *priori* group consists of 1 sample (the pervious 1st a *priori* groups in step 16a, c), 2nd a *priori* group consists of 1 sample (the pervious 2nd a *priori* groups in step 16a, c), the added 3rd a *priori* group is *L. pseudobrassicae* (3rd a *priori* group in steps 3, 7). CAP A5 resulted in acceptable mis-classification error (b). The molecular identification of 1st and 2_{nd} a *priori* groups revealed that they are *Rhodobium porosum* and *Aphis illinoisensis*, respectively.

(a)

(b)

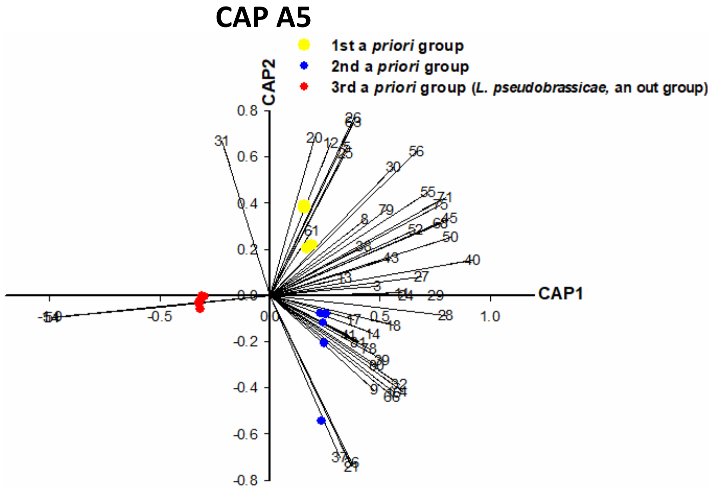

a priori groups		1	2	3	Total	%correct
Group	1	3	1	0	4	75.00%
Group	2	0	6	0	6	100.00%
Group	3	0	0	5	5	100.00%

Total correct = 14/ 15 = = 93.333%

Mis-classification error = 6.667%

(a) Simple scatter and vectors plots of CAP A4 resulted in clear visual distribution of the 3 a *priori* groups with their characteristic vectors. 1st a *priori* group: *R. porosum* (**yellow dots**) is isolated by vectors #26, #63, #20, #12, #25, #26, #30, #56, #61, and #63. 2nd a *priori* group: *A. illinoisensis* (**dark blue dots**) is isolated by vectors #4 #32, #66, #16, #36, #21, #37, #9, #39, and #80. The vectors of the out group: *L. pseudobrassicae* (**red dots**) were mentioned in step 11-f. (b) Mis-classification error is 6.67% (**red frame**).

19- 2nd a *priori* group in step 17 consists of 6 samples (the pervious 3rd, 4th, 5th, 6th, 7th, and 8th a *priori* groups in step 16a, c) and thus it was subjected to CAP B (1-3) analyses. CAP B1 resulted in a very good visual separation for 2nd and 4th a *priori* groups, while 1st and 3rd a *priori* groups formed a cluster and 5th and 6th a *priori* groups formed another cluster. This classification resulted in an error equal to 4.00%. The molecular identification of 2nd and 4th a *priori* groups revealed that both samples are *Aphis gossypii*.

(a)

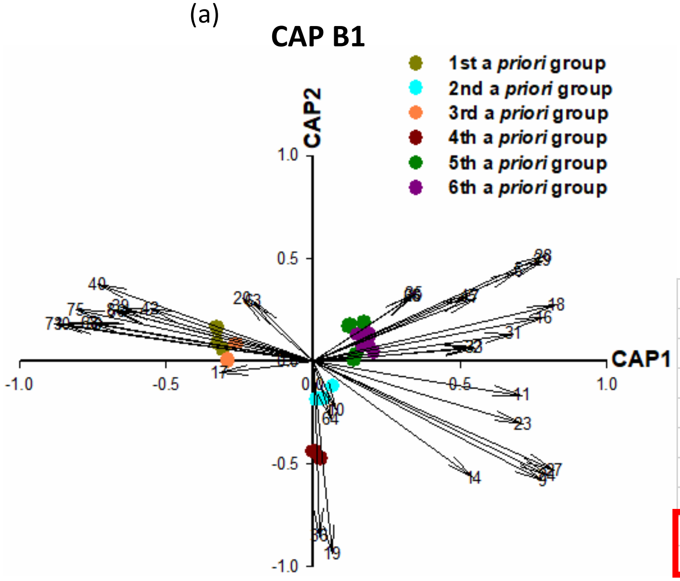

(b)

a *priori* groups		1	2	3	4	5	6	Total	%correct
Group	1	4	0	0	0	0	0	4	100.00%
Group	2	0	3	0	0	0	0	3	100.00%
Group	3	0	0	3	0	0	0	3	100.00%
Group	4	0	0	0	3	0	0	3	100.00%
Group	5	0	0	0	0	5	0	5	100.00%
Group	6	0	0	0	0	1	6	7	85.71%

Total correct = 24/ 25 = 96.000%
Mis-classification error = 4.000%

(a) Simple scatter and vectors plots of CAP B1 resulted in clear visual distribution of the 2 a *priori* groups with their characteristic vectors. 2nd a *priori* group: *A. gossypii*-lemon (**light blue dots**) is isolated by vectors #64, and #10. 4th a *priori* group: *A. gossypii*-mint (**dark red dots**) is isolated by vectors #19, and #36.

20- To separate the four a *priori* groups 1st, 3rd, 5th, and 6th in step 19, CAP B2 was performed. 1st a *priori* group (1st a *priori* group in step 20), 2nd (3rd a *priori* group in step 19), 3rd a *priori* group (5th a *priori* group in step 19), 4th a *priori* group (6th a *priori* group in step 19). CAP B2 separates 1st and 2nd a *priori* groups while 3rd and 4th a *priori* groups formed a cluster as it was observed in step 19. Both 1st and 2nd a *priori* groups were identified as *A. gossypii*.

(a)

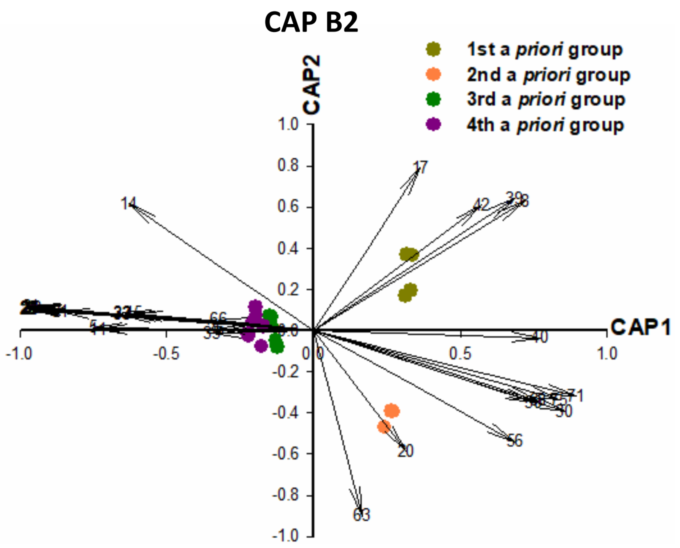

(b)

a priori groups		1	2	3	4	Total	%correct
Group	1	4	0	0	0	4	100.00%
Group	2	0	3	0	0	3	100.00%
Group	3	0	0	5	0	5	100.00%
Group	4	0	0	0	7	7	100.00%

Total correct = 19/ 19 = 100.000%
Mis-classification error = 0.000%

(a) Simple scatter and vectors plots of CAP B2 resulted in clear visual distribution of the 2 a *priori* groups with their characteristic vectors. 1st a *priori* group: *A. gossypii*-Hibiscus (**dark yellow dots**) is isolated by vectors #8, #39 and #42. 4th a *priori* group: *A. gossypii*-vinca (**orange dots**) is isolated by vector #20. (b) Mis-classification error is 0.0% (**red frame**).

21- Further CAP analysis was applied to separate 3rd and 4th a *priori* groups which were not separated in step 20. As CAP analysis cannot be applied on only two a *priori* groups, an outgroup was used to carry out CAP B3 which is *A. gossypii*-lemon (2nd a priori group in step 19). The 3 a *priori* groups are: 1st a *priori* group (3rd a *priori* group in step 20), 2nd a *priori* group (4th a *priori* group in step 20), and 3rd a *priori* group is *A. gossypii*-lemon (2nd a *priori* group in step 19). The last separated two a *priori* groups were identified by molecular identification as *A. gossypii*.

(a)

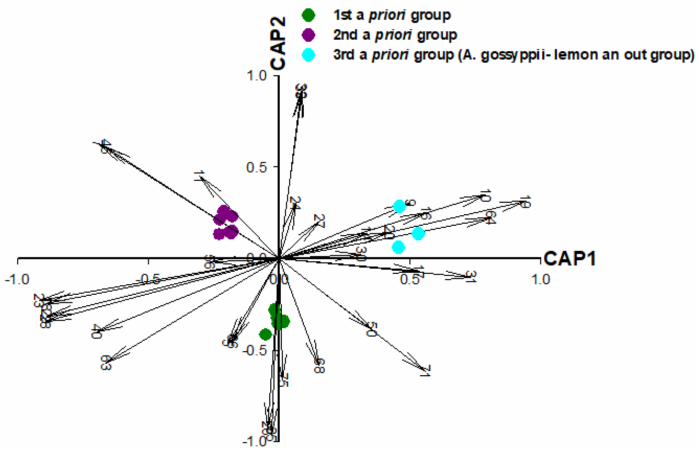

(b)

a priori groups		1	2	3	Total	%correct
Group	1	3	0	0	3	100.00%
Group	2	0	5	0	5	100.00%
Group	3	0	0	7	7	100.00%

Total correct = 15/ 15 = 100.000%
Mis-classification error = 0.000%

(a) Simple scatter and vectors plots of CAP B3 resulted in clear visual distribution of the 2 a *priori* groups with their characteristic vectors. 1st a *priori* group: *A. gossypii*-zucchini (**dark green dots**) is separated by vectors #35, #26, #75 and #5. 1st a *priori* group: *A. gossypii*-fresh feed (**dark pink dots**) is separated by vectors #7, #45, and #11. The vectors of the out group: *A. gossypii*-lemon (**light blue dots**) were mentioned in step 19-a. (b) Mis-classification error is 0.0% (**red frame**).

After the 21 steps, we concluded that the significant value of misclassification error that represents reliable separation is ≤ 7. This value will vary depending on the visual distribution of samples and the clarity of the vector(s) that characterize each sample. We found that the last six samples of aphid species collected from six different host plants identified chemically by CAP B1, CAPB2, and CAP B3 belong to the same genetic species which is *A. gossypii*. Thus, the differences between vectors that contributed to separate these six samples resulted from the host plants fed on by *A. gossypii* and not from *A. gossypii* itself. Going back to the CAP A4-to combine these six samples together **(light blue triangles**), we will find the chemical vectors that characterize *A. gossypii* regardless the host plants.

3. Supplementary Discussion

3.1 Chemotaxonomy of aphids.

Hydrocarbons, *n*-alkanes and methyl branched alkanes (Me-Alk), constitute often EBs to differentiate aphid species (see dichotomous key, Figure 10). However, EBs of *L. pseudobrassicae*, *H. setariae*, *A. nerii*, and *A. illinoisensis*, are isothiocyanates, aldehydes, terpenes, and esters, respectively. SEBs were essentially terpenes, alkenes, and ketones.Cuticular hydrocarbons can be used to to classify some aphids [2]. Six *A. gossypii* samples were identical in their COI gene but exhibited differences in their headspace profiles.

3.2 Hydrocarbon profiles of aphid's species upon cell damage.

The headspace compounds released by aphids after homogenization in liquid nitrogen were recorded by GC-MS. Compounds were identified by analysing their mass spectra and comparison of the mass spectra to the NIST library. CAP analysis was used to identify EB and SEBs.

CAP A2 indicated that a docosane and pentadecane are EBs and decane, 8-heptadecene and (*E*)-9-eicosene are SEBs of *E. lanigerum*. Based on the intensities and occurrence of headspace compounds (CAP A3), only one Me-Alk was considered as EB to characterize *A. craccivora* (3,8-dimethylundecane) and *M. rosae* (4,5-dimethylnonane). In the headspace of *A. punicae*, a mixture of linear and Me-Alks was detected as EB (tridecane, tetracosane, 4,7-dimethyl-undecane, 4,6-dimethyl-dodecane) and two as SEB (decane, nonadecane).

CAP A4 grouped six samples into an individual species, although the samples for this species were collected from six different host plants. Phylogenetic analysis identified all the six isolates as *A. gossypii* (Table S1).

The phylogenetic identification matched perfectly to the chemotaxonomic analysis because all six *A. gossypii* isolates were grouped in to one group based on the five alkanes as EB: 4,5-dimethylnonane, tetradecane, 2,6,10,15-tetramethyl-heptadecane, heptacosane, octacosane and the aphid pheromone (*E*)-β-farnesene (Figures 5, 10, 11). That the six isolates (Figure 5) were identified to belong to one species clearly proves that the influence from headspace compounds because of feeding on different host plants does not interfere with the species identification of *A. gossypii*.

Discriminant analysis (CAP A5, Figure 6) succeeded in separating *R. porosum* and *A. illinoisensis* by only one major hydrocarbon for each species: hexadecane (EB) and tetratetracontane respectively [3].

3.3 Alcohol profiles of aphid species.

1-Tetradecanol was identified as an EB of *E. lanigerum* (Figure 10, 11). Tetradecanol is has been found in many animals. [4]. In the *E. lanigerum* headspace profile, 1-tetradecanol was associated with the SEB n-decane (Figure 10).

3.4 Ketone profiles of aphid species.

Ketones constitute SEBs of *A. punicae* (6-methyl-5-hepten-2-one) and of *H. setariae* (2,6-di-*tert*.-butyl-*p*-benzoquinone) (Figure 10). Comparison of the headspace profile upon cell damage of *A. punicae* and *Tapinoma magnum* ants which are associated with *A. punicae* protecting the ants [5] (Figure S2): Thus headspace compounds of *A. punicae* (Figure S1A) may contain compounds from *T. magnum* (Figure S2B). The peak intensity of two ketones: 6-methyl-5-hepten-2-one [3] and 2-methyl-4-heptanone in *A. punicae* depended on how intensively *T. magnum* interacted with *A. punicae*, rendering the volatiles less suitable for a reliable biomarker.

It should be noted that H. setariae was collected in the absence of the black fungus, suggesting that the origin of 2,6-di-tert-butyl-p-benzoquinone was a green foxtail. The combination of the presence of the two VOCs: benzaldehyde as an EB, and nonanal as an SEB (Figure 10) supported that the collection time of *H. seteriae* was during severe aphid infestations.

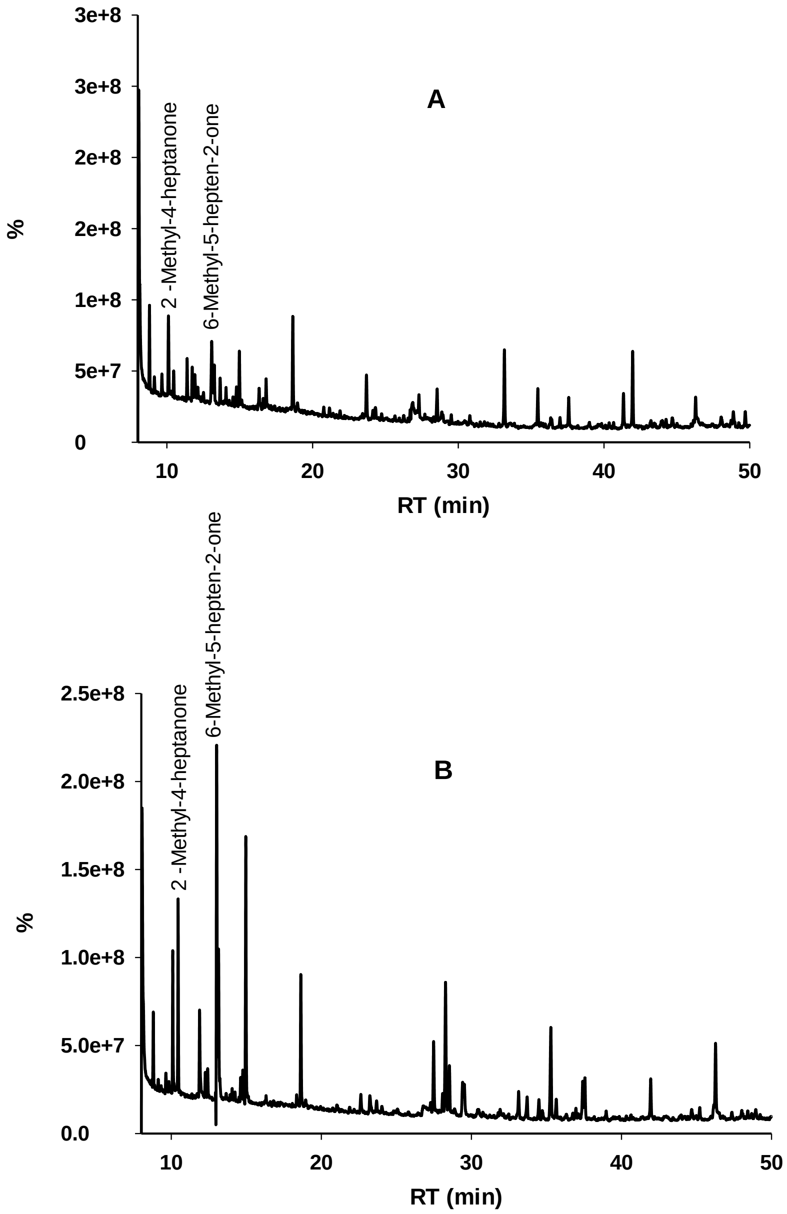

Figure S2: GC-MS chromatograms of headspace metabolites upon cell damage of the aphid *A. punicae* (A) and its associated ant *T. magnum* (B) suggesting that 2-methyl-4-heptanone and 6-methyl-5-hepten-2-one in the profile of *A. punicae* originates from its symbiotic ant *T. magnum*.

3.5. Ester and benzenoids in headspace profiles of aphids.

In the headspace of *A. punicae*, 1,1-diacetoxyethane and methyl pentacosanoate represented the primary EB and SEB, respectively (Figure 10). Plants are generally rich in esters, [6] and the extract of plant-derived-esters often have insecticidal or pesticide activities as sucking insects such as aphids [7]. reported cuticular surface lipids/waxes of ants (e.g., *Pogonomyrmex barbatu*) as another source of esters, with C19–C31 carbon range.

In headspace profile of *A. illinoisensis*, 2,2,4-trimethylpentane-1,3-diyl bis(2-methylpropanoate) (TXIB), and benzenoids (i.e., Bnz-16, Bnz-32, and mesitylene) were identified as EBs, while methyl hexadecanoate was detected as SEB (Figure 10). Whitehead and Douglas (1993) showed that bacteria represent 2–5 % of the aphid biomass [8]. The bacterial population increases progressively with aphid age, to offered immediate fitness benefits under environmental stress and the bacteria also offer an immediate protection against parasitoids [9].

In this study, TXIB, eucalyptol, mesitylene and other benzenoids reflect undefined post-cellular damage – reformulate EBs from A. Illinoisensis.It is therefore likely that the coexistence of these biomarkers is associated with intra- or extracellular symbiotes[10] . Accordingly, the existence of tetratetracontane as a SEB is suspected to orginate from associated microbial symbionts. Moreover, MeSA and methyl hexadecanoate as SEBs as plant defense compounds indicate that the grape leaves and stems were in the advanced stage of infestation by *A. illinoisensis* aphids, which were in their late growth stage with high biomass of bacterial symbionts [8].

3.6. Plant defense related compounds in aphids.

The phytohormone MeSA was identified as an EB of *A. nerii* collected from oleander (Apocynaceae) because of its abundance in the aphids' headspace (Figure 10).. MeSA is a plant defense response against sucking-piercing insects, such as aphids [11]. Apart from its toxicity [11], MeSA serves as volatile cue to warn other plants of pathogen attacks [12]Numerous plants produce MeSA in minute amounts, except for the Apocynaceae family, which produce large amounts of MeSA[13].Many other factors influence MeSA emissions (e.g., plant species and age). However, [14] in aphid-infested plants, MeSA is along with the aphid alarm pheromone EBF significantly increased.

MeSA is present in the headspace profile of *A. nerii*, *E. langerium*, *A. illinoisensis*, and *A. gossypii*-Hibiscus (Table S2). It represents an EB in *A. nerii*, and a SEB in *E. langerium*. Based on the phylogenetic tree, *A. nerii* and *A. illinoisensis* belong to different clades, although they share MeSA as either an EB (*A. nerii*) or SEB (*A. illinoisensis*). Among the 10 species identified by phylogeny (Figure S1), *A. nerii* and *E. lenigerum* belonged to same clade, where *A. nerii, H. setariae*, and *E. Lenigerum* showed a strongly supported cluster (PP = 88%). Thus, MeSA can be used for diagnoses of plant infestation, but not species identification.

According to the classification of MeSA based on CAP analysis (Figure 10), as an EB for *A. nerii*, SEB for *A. illinoisensis*, and a headspace compound of *E. lanigerum*, the descending order of aphid density in infested plants was: *A. nerii* > *A. illinoisensis* > *E. lanigerum*.

For *H. setariae*, the main headspace biomarkers (bio14, bio42) detected were benzaldehyde and caprolactam respectively both of which occured in high concentration. Both compounds are also found from the infested plant (green foxtail plant)[15] Caprolactam was degraded from protein in insects as a response to chemical pesticides and mechanical stressors[16]. Benzaldehyde is a common component of plant VOCs, and is intended to attract various pest species[17].The absence of mVOCs in biomarker patterns of *H. setariae* led us to the prediction that tetratetracontane as an SEB likely originates from the host plant (green foxtail plant) [15].

3.7 Rapid diagnosis of aphid species by GC-MS and CAP analysis.

Table S3: Compounds (n= 11) released into the headspace upon cell damage of the unknown green aphid collected from sow-thistle (Asteraceae) in February 2022, Taif Governorate (Saudi Arabia). Metabolites were extracted by CLS and analyzed by GC-MS.

Biomarkers	RT (min)	Compounds
Bio4	10:26	α-Thujene
Bio82	10:53	3-Carene
Bio10	12:24	β-Thujene
Bio84	12:54	Ψ-Limonene
Bio14	13:26	Bnz -14
Bio85	14:39	α-Pinene
Bio20	14:96	D-Limonene
Bio21	15:08	Eucalyptol
Bio86	16:65	γ-Terpinene
Bio30	18:62	4,5-Dimethyl-nonane
Bio56	35:46	(*E*)-β-Farnesene

3.8. Comparison of headspace collection methods.

In order to investigate to which extent, the headspace sampling influences the GC-MS chromatogrammes and thus the data analysis instead of CLS of activated charcoal (3 mg, CLS-charcoal). Opened loop striping (OLS) using divinylbenzene polymer (5 mg, OLS-DVBP) was tested.

Headspace volatiles were collected using a dynamic 'push–pull' pump system. The pushed airflow was set to 1 l·min^{-1} to carry air through the collection chamber and the pulled air

flow was set to 0.5 L·min^{-1}. The air entering the collection chamber (5 ml vial) was cleaned by an activated charcoal filter to avoid any contamination. A 30 mg HayeSep Q tube (Volatile Assay Systems, Rensselaer, New York, USA), which is composed of spherical particles of a divinylbenzene polymer (DVB), was placed at the outlet of the vial to trap the volatile compounds. Before use the HayeSep Q tubes were cleaned by ethyl acetate (x ml, HPLC grade, Aldrich, Germany), and a purified inert nitrogen gas regulated to between 10 and 20 psi to push the solvent through the trap until the adsorbent was dry (x min). DVB traps were eluted by 60 µl ethylacetate (HPLC grade, Aldrich, Germany). The elute was collected in 0.1 ml inserts and kept in 1.5 mL autosampler vials that were closed tightly (VWR, Germany). The vials were stored at -80 °C until they were analysed by GC-MS.

The comparison of both methods clearly indicates that CLS-charcoal collection was superior to OLS-DVBP because much more compounds with higher peak intensities could be observed when CLS-charcoal was used (Figure S2). Thus, CLS-charcoal was used as method of choice for the headspace collection.

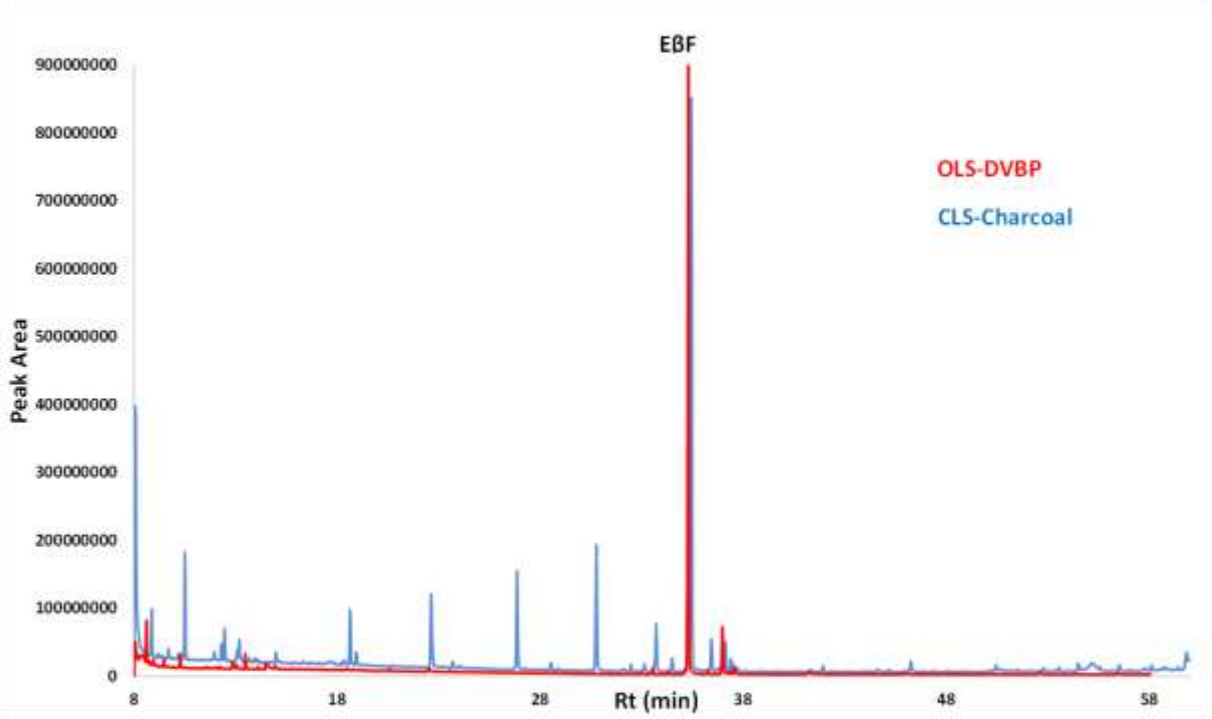

Figure S3: GC-MS chromatograms of headspace profile extracted from crushed *M. rosae* by CLS-charcoal (blue) and OLS-DVBP (red).

3.9. GC chromatogram of multiple individual's vs one individual of aphid species.

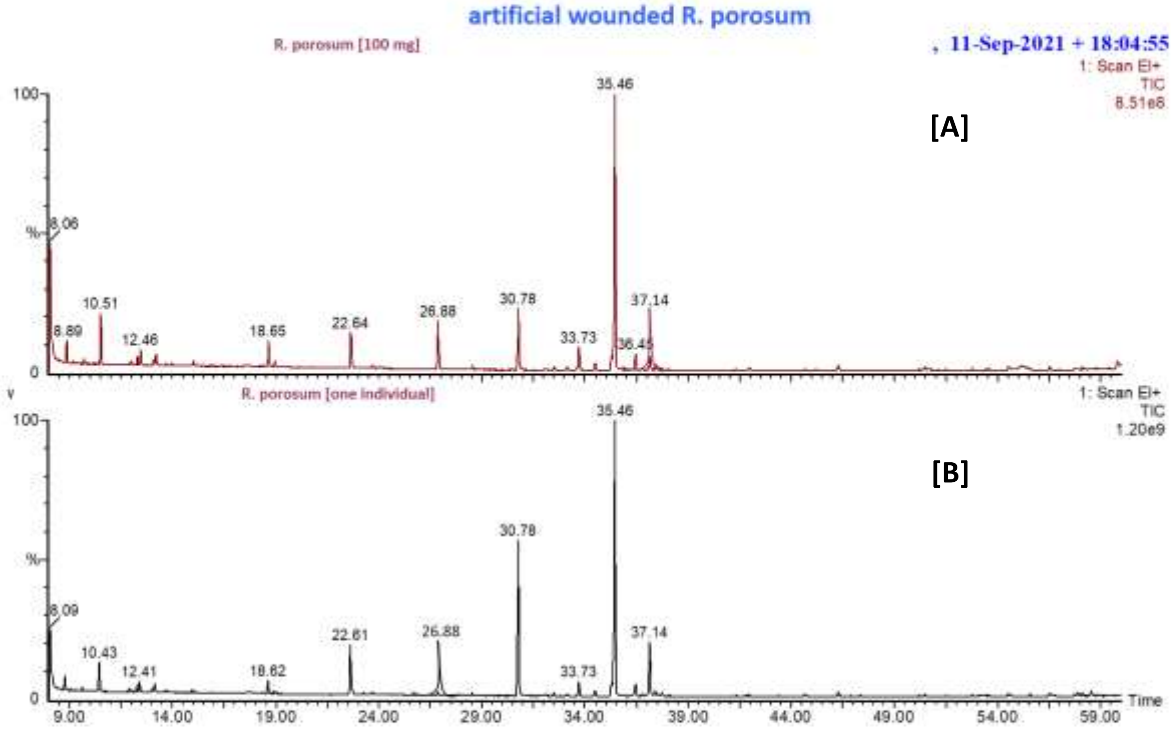

Figure S4: GC chromatogram of *R. porosum* (A) multiple individuals, (B) one individual. The chromatograms verify the reproducibility of chemotaxonomy approach.

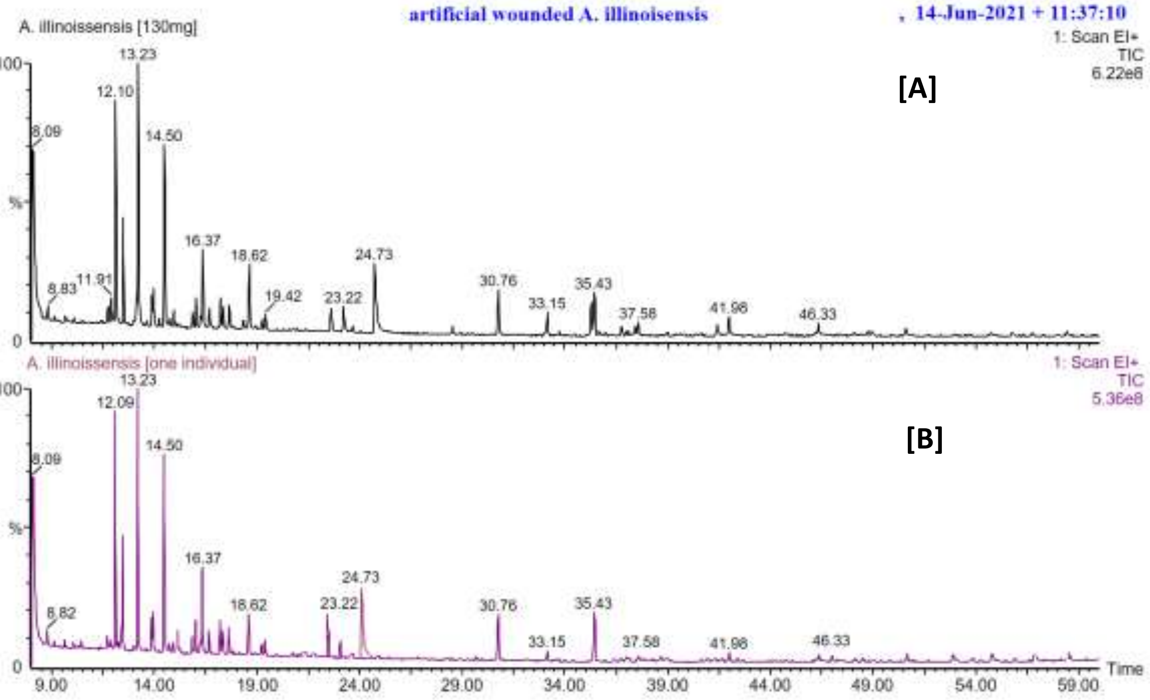

Figure S5: GC chromatogram of *A. Illinoisensis*. (A) multiple individuals, (B) one individual. The chromatograms verify the reproducibility of chemotaxonomy approach.

References

1. Anderson, M. and T. Willis, *Canonical analysis of principal coordinates: A useful method of constrained ordination for ecology.* Ecology, 2003. **84**: p. 511-525.
2. Raboudi, F., et al., *Aphid species identification using cuticular hydrocarbons and cytochrome b gene sequences.* Journal of Applied Entomology, 2005. **129**: p. 75-80.
3. Kergunteuil, A., et al., *Characterizing Volatiles and Attractiveness of Five Brassicaceous Plants with Potential for a 'Push-Pull' Strategy Toward the Cabbage Root Fly, Delia radicum.* Journal of chemical ecology, 2015. **41**.
4. Kochansky, J., J.R. Aldrich, and W.R. Lusby, *Synthesis and pheromonal activity of 6,10,13-trimethyl-1-tetradecanol for predatory stink bug,Stiretrus anchorago (Heteroptera: Pentatomidae).* Journal of Chemical Ecology, 1989. **15**(6): p. 1717-1728.
5. Vela, J., et al., *Aphids and Ants, Mutualistic Species, Share a Mariner Element with an Unusual Location on Aphid Chromosomes.* Genes (Basel), 2021. **12**(12).
6. Beekwilder, J., et al., *Functional Characterization of Enzymes Forming Volatile Esters from Strawberry and Banana.* Plant Physiology, 2004. **135**(4): p. 1865-1878.
7. Ahmed, Q., et al., *Evaluation of Aphicidal Effect of Essential Oils and Their Synergistic Effect against Myzus persicae (Sulzer) (Hemiptera: Aphididae).* Molecules, 2021. **26**(10).
8. Whitehead, L.F. and A.E. Douglas, *Populations of symbiotic bacteria in the parthenogenetic pea aphid (<i>Acyrthosiphon pisum</i>) symbiosis.* Proceedings of the Royal Society of London. Series B: Biological Sciences, 1993. **254**(1339): p. 29-32.
9. Pons, I., et al., *New Insights into the Nature of Symbiotic Associations in Aphids: Infection Process, Biological Effects, and Transmission Mode of Cultivable Serratia symbiotica Bacteria.* Appl Environ Microbiol, 2019. **85**(10).
10. Fukatsu, T., *Secondary intracellular symbiotic bacteria in aphids of the genus Yamatocallis (Homoptera: Aphididae: Drepanosiphinae).* Appl Environ Microbiol, 2001. **67**(11): p. 5315-20.
11. Ninkovic, V., et al., *Effects of Methyl Salicylate on Host Plant Acceptance and Feeding by the Aphid Rhopalosiphum padi.* Front Plant Sci, 2021. **12**: p. 710268.
12. Shulaev, V., P. Silverman, and I. Raskin, *Airborne signalling by methyl salicylate in plant pathogen resistance.* Nature, 1997. **385**(6618): p. 718-721.
13. Singewar, K., M. Fladung, and M. Robischon, *Methyl salicylate as a signaling compound that contributes to forest ecosystem stability.* Trees, 2021. **35**.
14. Kivimäenpää, M., et al., *Methyl Salicylate and Sesquiterpene Emissions Are Indicative for Aphid Infestation on Scots Pine.* Forests, 2020. **11**: p. 573.
15. Guo, K., et al., *Transcriptomic responses of three aphid species to chemical insecticide stress.* Science China Life Sciences, 2017. **60**.
16. Zhang, Z., et al., *iPathCons and iPathDB: an improved insect pathway construction tool and the database.* Database : the journal of biological databases and curation, 2014. **2014**.
17. Zhang, H., et al., *Identification of VOCs in essential oils extracted using ultrasound- and microwave-assisted methods from sweet cherry flower.* Scientific Reports, 2021. **11**.
